# Trends in the Use of Opioids vs Nonpharmacologic Treatments in Adults With Pain, 2011-2019

**DOI:** 10.1001/jamanetworkopen.2022.40612

**Published:** 2022-11-07

**Authors:** Kevin T. Pritchard, Jacques Baillargeon, Wei-Chen Lee, Mukaila A. Raji, Yong-Fang Kuo

**Affiliations:** 1 Department of Nutrition, Metabolism, and Rehabilitation Sciences, School of Public and Population Health, University of Texas Medical Branch, Galveston; 2 Department of Epidemiology, School of Public and Population Health, University of Texas Medical Branch, Galveston; 3 Department of Internal Medicine, University of Texas Medical Branch, Galveston; 4 Department of Biostatistics and Data Science, School of Public and Population Health, University of Texas Medical Branch, Galveston

## Abstract

**Importance:**

Chronic pain prevalence among US adults increased between 2010 and 2019. Yet little is known about trends in the use of prescription opioids and nonpharmacologic alternatives in treating pain.

**Objectives:**

To compare annual trends in the use of prescription opioids, nonpharmacologic alternatives, both treatments, and neither treatment; compare estimates for the annual use of acupuncture, chiropractic care, massage therapy, occupational therapy, and physical therapy; and estimate the association between calendar year and pain treatment based on the severity of pain interference.

**Design, Setting, and Participants:**

A serial cross-sectional analysis was conducted using the nationally representative Medical Expenditure Panel Survey to estimate the use of outpatient services by cancer-free adults with chronic or surgical pain between calendar years 2011 and 2019. Data analysis was performed from December 29, 2021, to August 5, 2022.

**Exposures:**

Calendar year (2011-2019) was the primary exposure.

**Main Outcomes and Measures:**

The association between calendar year and mutually exclusive pain treatments (opioid vs nonpharmacologic vs both vs neither treatment) was examined. A secondary outcome was the prevalence of nonpharmacologic treatments (acupuncture, chiropractic care, massage therapy, occupational therapy, and physical therapy). All analyses were stratified by pain type.

**Results:**

Among the unweighted 46 420 respondents, 9643 (20.4% weighted) received surgery and 36 777 (79.6% weighted) did not. Weighted percentages indicated that 41.7% of the respondents were aged 45 to 64 years and 55.0% were women. There were significant trends in the use of pain treatments after adjusting for demographic factors, socioeconomic status, health conditions, and pain severity. For example, exclusive use of nonpharmacologic treatments increased in 2019 for both cohorts (chronic pain: adjusted odds ratio [aOR], 2.72; 95% CI, 2.30-3.21; surgical pain: aOR, 1.53; 95% CI, 1.13-2.08) compared with 2011. The use of neither treatment decreased in 2019 for both cohorts (chronic pain: aOR, 0.43; 95% CI, 0.37-0.49; surgical pain: aOR, 0.59; 95% CI, 0.46-0.75) compared with 2011. Among nonpharmacologic treatments, chiropractors and physical therapists were the most common licensed healthcare professionals.

**Conclusions and Relevance:**

Among cancer-free adults with pain, the annual prevalence of nonpharmacologic pain treatments increased and the prevalent use of neither opioids nor nonpharmacologic therapy decreased for both chronic and surgical pain cohorts. These findings suggest that, although access to outpatient nonpharmacologic treatments is increasing, more severe pain interference may inhibit this access.

## Key Points

**Question:**

What are the annual trends in access to pharmacologic and nonpharmacologic pain treatments among cancer-free US adults with chronic or surgical pain?

**Findings:**

In this serial cross-sectional study of Medical Expenditure Panel Survey data from 2011 to 2019 with 46 420 respondents, the prevalence of outpatient nonpharmacologic treatments surpassed prescription opioid use for chronic, but not surgical, pain.

**Meaning:**

The findings of this study suggest that nonpharmacologic pain treatments are increasing in prevalence following government policy and practice guidelines; however, barriers to accessing some services may still exist.

## Teaser

This cross-sectional study examines changes in the use of opioid treatment and nonpharmacologic treatment in the US from 2011 to 2019.

## Introduction

Chronic pain prevalence among US adults was 19.0% in 2010,^1^ increasing to estimated rates of 20.4% in both 2016^2^ and in 2019.^3^ Annual pain expenses—compared with the average person without pain—range between $261 billion and $300 billion for increased health care use and $299 billion and $355 billion for lost productivity, exceeding the costs of heart disease, cancer, or diabetes.^4^ In response, Healthy People 2030 seeks to reduce the prevalence of adult chronic pain that interferes with daily activities from 6.9% to 6.4%.^5^

A shared vision for future activity-limiting pain treatment is to prioritize interventions that consider the biopsychosocial nature of pain.^6,7,8^ Prescription opioids were used by 22.1% of US adults with chronic pain during 2019,^9^ but opioid analgesics are associated with a heightened risk of adverse events including falls,^10^ misuse or diversion,^11^ preventable hospital admissions, and overdose mortality.^12,13,14,15^ Despite earlier reports that low-risk nonpharmacologic interventions can simultaneously reduce pain and improve function,^16,17,18,19^ to our knowledge, no study has examined access to nonpharmacologic interventions used by cancer-free adults with or without surgery. We analyzed surgical pain separately to account for different pain management guidelines^20,21^ and because the National Pain Strategy recognizes that when opioids are appropriately prescribed, they can be effective for postsurgical pain.^8^ Trends in opioid use are well explicated,^9,22,23^ yet the Centers for Disease Control and Prevention (CDC) reported a need to contextualize treatment trends based on pain interference severity.^9^ Previous studies often measured access to intervention techniques instead of to the licensed health care professionals^24^ who treat pain in the clinical setting.^22,23,25,26,27^ Therefore, we defined nonpharmacologic treatments based on a policy brief^24^ that identified the licensed health care professionals (acupuncturists, chiropractors, massage therapists, occupational therapists, and physical therapists) specialized in treating pain.^18,19^ Operationalizing the workforce, instead of intervention techniques, helps establish a clear process for patient referral.

We aimed to (1) describe annual trends in the mutually exclusive use of prescription opioids, nonpharmacologic treatments, both treatments, and neither treatment; (2) describe annual trends in the use of various nonpharmacologic treatments; (3) examine whether calendar year was associated with treatment type after adjusting for demographic characteristics, socioeconomic status, health conditions, and pain interference severity; and (4) determine whether the annual use of each treatment varied by pain interference severity. We hypothesized that the annual prevalence of nonpharmacologic treatments would increase and depend on pain interference severity due to the 2014 Drug Enforcement Administration policy rescheduling hydrocodone^28^ and the 2016 CDC guideline for prescribing opioids for chronic pain, which aimed to curtail prescription opioid use and encourage nonpharmacologic treatments.^20^

## Methods

### Data Sources

A serial cross-sectional design and the Agency for Healthcare Research and Quality Medical Expenditure Panel Survey–Household Component (MEPS) were used to describe period prevalence for pain treatments during 2011-2019. The University of Texas Medical Branch Institutional Review Board determined that this study was not human participant research. In accordance with 45 CFR §46, informed consent was not needed. We followed the Strengthening the Reporting of Observational Studies in Epidemiology (STROBE) reporting guideline.

Beginning in 1996, MEPS targets the noninstitutionalized US civilian population and annually obtains subsamples from the total primary sampling units in the National Center for Health Statistics’ National Health Interview Survey. Racial and ethnic minority households were oversampled to produce unbiased national and regional estimates. Response rates ranged from 73.2% to 82.1% for the National Health Interview Survey and from 62.9% to 68.8% for MEPS.^29^ Survey weights are derived from the National Health Interview Survey household weight, adjusted for nonresponse at the household and person levels, and undergo poststratification raking. Survey weights account for unequal probabilities of selection into MEPS, nonresponse, attrition, and generalizability to the target population. Data came from the following 7 publicly available Household Component files: full year consolidated, medical conditions, prescribed medicines, and emergency department, inpatient, office-based, and outpatient visits.

### Participant Cohort

The cohort included cancer-free adults with chronic (n = 36 777) or surgical (n = 9643) pain (eFigure 1 in the Supplement). Respondents were excluded if they were younger than age 18 years (27.3%), had a history of cancer (6.0%) according to the year’s medical conditions files, or had no qualifying 3-digit *International Classification of Diseases, Ninth Revision* (*ICD-9*) or *International Statistical Classification of Diseases, Tenth Revision* (*ICD-10*) code for chronic (eTable 1 in the Supplement) or surgical (eTable 2 in the Supplement) pain (75.7%). Because cancer diagnoses require different pain treatments and predispose patients to prolonged opioid use,^20,30^ we excluded using 3-digit *ICD-9* codes (140-239) in 2011-2015 and *ICD-10* codes (C00-D49) in 2016-2019. Detailed information on the cohort validity,^31^ reliability,^32^ and efforts to reduce misclassification bias appears in the footnotes of eTables 1 and 2 in the Supplement.

### Outcomes

Access, instead of dosage, was measured to expand upon the Agency for Healthcare Research and Quality National Healthcare Quality and Disparities Report, which annually tracks access to common health care services.^33^ The primary outcomes included 4 mutually exclusive types of pain treatments: (1) opioids only, (2) nonpharmacologic alternatives only, (3) both opioid and nonpharmacologic alternatives, and (4) neither opioids nor nonpharmacologic therapy. Because about 8.5%^32,34^ of opioid prescriptions in MEPS and 8.1%^35^ of chiropractic or massage therapy in the National Health Interview Survey are due to nonpain conditions, we only included treatments that occurred during health care visits for the chronic and surgical pain *ICD-9* and *ICD-10* codes used for cohort inclusion. Prescription opioid use estimated the annual rate of receiving at least 1 opioid prescription and was identified by Cerner Multum Lexicon therapeutic drug class codes: 60 narcotic analgesics and 191 narcotic analgesic combinations.^23,36,37,38^ MEPS staff verified prescriptions with the respondent’s pharmacy, achieving a 73.0% to 87.8% pair-level completion rate in 2011-2019.^38,39^ The use of nonpharmacologic alternatives estimated the annual prevalence of at least 1 outpatient or office-based visit with any of the following health care professionals: acupuncturist, chiropractor, massage therapist, occupational therapist, and/or physical therapist. In 2017, MEPS coded occupational therapy (OT) and physical therapy (PT) as a single profession; therefore, we grouped these professions for all years designating OT/PT as OT and/or PT.

### Primary Exposure and Covariates

The primary independent variable was self-reported year of health service use (2011-2019). Pain interference was ascertained via the self-administered questionnaire, which asked respondents, “During the past 4 weeks, how much did pain interfere with your normal work (including both work outside the home and housework),” using the Veterans RAND 12 Item Health Survey (a little bit, moderately, quite a bit, extremely).^40^ Covariates, defined in Table 1, included age, sex, self-reported race and Hispanic ethnicity, educational level, family income as percentage of the poverty line,^9^ Census region,^10^ insurance type,^41^ and number of comorbidities.

**Table 1.** Demographic Characteristics of the 2011-2019 MEPS Study Population

Characteristic	Chronic pain		Surgical pain		Total		Design effect size^a^
	Unweighted, No.(n = 36 777)	Weighted, % (95% CI) (n = 442 813 500)	Unweighted, No. (n = 9643)	Weighted, % (95% CI) (n = 116 952 068)	Unweighted, No. (N = 46 420)	Weighted, % (95% CI) (N = 539 765 568)	
Year of pain treatment							
2011	3727	9.8 (9.3-10.3)	1143	11.8 (11.0-12.7)	4870	10.2 (9.8-10.7)	2.77
2012	4319	9.9 (9.4-10.4)	1215	11.5 (10.6-12.3)	5534	10.3 (9.8-10.7)	2.80
2013	4507	11.6 (11.1-12.1)	1248	12.6 (11.7-13.4)	5755	11.8 (11.4-12.3)	2.25
2014	4401	12.6 (12.0-13.1)	1228	12.9 (12.0-13.8)	5629	12.7 (12.1-13.2)	2.84
2015	4668	12.9 (12.2-13.5)	1299	14.4 (13.5-15.3)	5967	13.2 (12.6-13.7)	3.14
2016	4857	13.2 (12.7-13.8)	1006	10.5 (9.7-11.2)	5863	12.6 (12.1-13.2)	2.87
2017	4486	12.8 (12.1-13.4)	873	9.0 (8.2-9.8)	5359	11.9 (11.4-12.5)	3.21
2018	3081	8.9 (8.2-9.6)	857	8.8 (7.9-9.7)	3938	8.9 (8.2-9.5)	6.34
2019	2731	8.3 (7.8-8.9)	774	8.6 (7.7-9.5)	3505	8.4 (7.8-8.9)	4.54
Pain interference^b^							
Not at all	12 839	36.8 (36.0-37.7)	2775	30.6 (29.4-31.8)	15 614	35.5 (34.7-36.2)	2.87
A little bit	10 570	29.9 (29.2-30.5)	2353	26.1 (24.9-27.3)	12 923	29.1 (28.5-29.6)	1.70
Moderately	5624	14.8 (14.3-15.3)	1595	16.5 (15.6-17.4)	7219	15.2 (14.8-15.6)	1.74
Quite a bit	5320	12.7 (12.2-13.3)	1938	18.1 (17.2-19.0)	7258	13.9 (13.4-14.4)	2.43
Extremely	2424	5.7 (5.4-6.0)	982	8.7 (8.0-9.4)	3406	6.4 (6.0-6.7)	2.06
Pain intervention type							
Neither treatment^c^	23 262	60.4 (59.5-61.4)	4864	49.2 (47.8-50.5)	28 126	58.0 (57.2-58.8)	3.55
Opioid prescription only	5159	12.8 (12.2-13.4)	2572	25.5 (24.3-26.7)	7731	15.5 (14.9-16.2)	3.72
Nonpharmacologic treatment only	7119	23.1 (22.2-24.0)	1089	12.6 (11.7-13.5)	8208	20.8 (20.0-21.6)	4.49
Both opioid prescription and nonpharmacologic treatment	1237	3.7 (3.4-3.9)	1118	12.7 (11.9-13.6)	2355	5.6 (5.3-5.9)	2.10
Age, y							
18-44	12 603	34.9 (33.8-35.9)	2555	25.7 (24.5-27.0)	15 158	32.9 (31.9-33.8)	4.90
45-64	15 672	42.2 (41.3-43.1)	3838	39.6 (38.2-41.0)	19 510	41.7 (40.8-42.5)	3.39
≥65	8502	22.9 (22.1-23.8)	3250	34.7 (33.2-36.1)	11 752	25.5 (24.6-26.3)	4.42
Sex							
Female	20 978	54.2 (53.5-54.9)	5889	58.0 (56.8-59.2)	26 867	55.0 (54.4-55.7)	1.92
Male	15 799	45.8 (45.1-46.5)	3754	42.0 (40.8-43.2)	19 553	45.0 (44.3-45.6)	1.92
Race and ethnicity							
American Indian or Alaska Native or unspecified	1386	3.4 (3.0-3.9)	355	3.2 (2.6-3.8)	1741	3.4 (2.9-3.8)	7.34
Asian or Native Hawaiian or Other Pacific Islander	2311	5.2 (4.6-5.7)	301	2.3 (1.7-2.9)	2612	4.5 (4.0-5.1)	7.73
Black	6906	11.1 (10.2-12.0)	1686	9.6 (8.6-10.5)	8592	10.7 (9.9-11.6)	9.21
White	26 174	80.3 (79.1-81.5)	7301	85.0 (83.7-86.2)	33 475	81.3 (80.2-82.5)	10.19
Hispanic							
No	28 895	87.5 (86.4-88.6)	7990	90.3 (89.1-91.5)	36 885	88.1 (87.0-89.2)	13.30
Yes	7882	12.5 (11.4-13.6)	1653	9.7 (8.5-10.9)	9535	11.9 (10.8-13.0)	13.30
Educational level							
No degree	6951	12.5 (11.9-13.2)	1650	12.1 (11.3-13.0)	8601	12.5 (11.8-13.1)	4.18
GED or high school diploma	15 328	41.4 (40.5-42.4)	4142	42.3 (41.0-43.6)	19 470	41.6 (40.8-42.5)	3.81
Associate, tech, or vocational degree	5066	14.7 (14.1-15.3)	1490	16.0 (15.0-17.0)	6556	15.0 (14.4-15.6)	3.15
Bachelor's degree	5749	18.9 (18.2-19.7)	1424	17.6 (16.4-18.7)	7173	18.6 (17.9-19.3)	3.91
Master's or doctoral degree	3433	11.8 (11.1-12.6)	881	11.5 (10.5-12.4)	4314	11.8 (11.1-12.5)	5.53
Unknown	250	0.5 (0.4-0.6)	56	0.5 (0.3-0.7)	306	0.5 (0.4-0.6)	2.24
Family income as % of poverty line^d^							
Poor/negative	6998	12.8 (12.1-13.5)	1825	12.4 (11.5-13.4)	8823	12.7 (12.1-13.4)	4.63
Near poor	2174	4.5 (4.1-4.8)	580	4.6 (4.1-5.1)	2754	4.5 (4.2-4.8)	2.07
Low income	5677	12.6 (12.1-13.1)	1441	13.0 (12.2-13.8)	7118	12.7 (12.2-13.1)	2.18
Middle income	10 178	27.7 (26.9-28.4)	2628	27.0 (25.9-28.2)	12 806	27.5 (26.9-28.2)	2.67
High income	11 750	42.5 (41.3-43.7)	3169	42.9 (41.4-44.5)	14 919	42.6 (41.5-43.7)	6.29
Census region							
Northeast	6126	17.7 (16.5-19.0)	1672	18.5 (16.6-20.4)	7798	17.9 (16.6-19.2)	13.68
Midwest	7845	22.4 (21.0-23.8)	2334	24.8 (23.2-26.4)	10 179	22.9 (21.6-24.3)	12.34
South	12 848	34.6 (33.0-36.2)	3565	36.5 (34.4-38.6)	16 413	35.0 (33.4-36.6)	13.64
West	9958	25.2 (23.7-26.8)	2072	20.2 (18.8-21.7)	12 030	24.2 (22.8-25.5)	12.36
Insurance coverage							
None	3828	8.0 (7.5-8.5)	440	3.6 (3.1-4.0)	4268	7.0 (6.6-7.5)	3.26
Private	21 344	67.5 (66.4-68.5)	5703	67.3 (65.8-68.8)	27 047	67.5 (66.5-68.5)	5.51
Public only	11 605	24.5 (23.5-25.5)	3500	29.1 (27.8-30.5)	15 105	25.5 (24.6-26.4)	5.43
No. of comorbidities^e^							
0	10 350	29.2 (28.3-30.1)	1729	18.0 (16.9-19.2)	12 079	26.8 (26.0-27.5)	3.69
1	8693	24.9 (24.2-25.7)	1909	20.8 (19.7-21.9)	10 602	24.0 (23.4-24.7)	2.74
2	7112	19.3 (18.6-20.0)	1903	21.1 (19.9-22.4)	9015	19.7 (19.1-20.3)	2.99
≥3	10 622	26.6 (25.7-27.5)	4102	40.0 (38.7-41.3)	14 724	29.5 (28.7-30.3)	4.04

Abbreviations: GED, general educational development; MEPS, Medical Expenditure Panel Survey–Household Component.

^a^ The average design effect for variables in this study is 4.97. Given this design effect and a nominal sample size of 46 420, the appropriate sample size for power analyses would be 9340. Taylor series linearization was used in all analyses to adjust variance estimation in accordance with these design effects.

^b^ Pain interference was defined as how often pain interferes with work or daily life using the Veterans RAND 12 Item Health Survey.

^c^ The neither treatment group used neither opioids nor nonpharmacologic therapy. The rates of other pharmacologic treatments known to reduce pain that were used by those in the neither treatment group can be viewed in eTable 4 in the Supplement.

^d^ Family income determined as percentage of poverty line (lowest income/negative [<100%], near lowest income [100% to <125%], low income [125% to <200%], middle income [200% to <400%], and high income [≥400%]).

^e^ Self-reported comorbidities included hypertension, coronary heart disease, high cholesterol level, emphysema, bronchitis, diabetes, arthritis, asthma, or stroke.

### Statistical Analysis

Data analysis was conducted from December 29, 2021, to August 5, 2022. All statistical analyses were stratified by pain type. To test the null hypothesis that the chronic and surgical pain cohorts had similar covariate distributions, we used Rao-Scott χ^2^ goodness-of-fit tests and 95% CIs. A supplemental analysis compared our included study population against our source population, defined as all noninstitutionalized US adults.

Descriptive analysis illustrated the annual prevalence of health service use by dividing 4 mutually exclusive outcomes by the number of respondents in each year’s cohort. A more detailed analysis illustrated the annual prevalence of respondents who accessed any acupuncturist, chiropractor, massage therapist, occupational therapist, or physical therapist. Weighted estimates are reported with 95% Clopper Pearson CIs.

Multivariable logistic regression models were used to permit within-group interpretation for the adjusted association between calendar year and each outcome. Owing to an increased type 1 error when conducting multiple logistic regressions, a sensitivity analysis using multinomial regression was conducted to verify robust annual trends and permit between-treatment group interpretations. No evidence of multicollinearity was found (*r* < 0.34). Overall linear trend tests were used to test the hypothesis of an association between calendar year and mutually exclusive pain treatment. An interaction between pain interference severity and calendar year was tested as an a priori hypothesis to determine whether annual treatment use varied by pain severity. Design-adjusted Wald χ^2^ analysis was used to assess model fit and test single-coefficient, multiparameter, and first-order interaction inferences.

To account for the complex survey design of MEPS, all analyses were weighted by the person-level, self-administered questionnaire weight. Complete case analysis was used because this survey weight accounted for unit nonresponse and MEPS imputations accounted for item nonresponse. Design-adjusted SEs were calculated with Taylor series linearization using sampling strata and cluster variables.^42^ All analyses were 2-sided with significance set at *P* < .05. Data management and analyses were conducted using SAS statistical software, version 9.4 (SAS Institute Inc).

## Results

### Cohort Characteristics

Among the unweighted 46 420 respondents, 9643 (20.4% weighted) received surgery and 40 602 (79.6% weighted) did not. Based on design-adjusted percentages, the total cohort tended to be aged 45 to 64 years (41.7%) and female (55.0%), have a general educational development or high school diploma (41.6%), have a high income (42.6%), reside in the southern US (35.0%), have no pain interference (35.5%), and use neither treatment (58.0%). The race and ethnicity categories of the population were as follows: American Indian/Alaska Native/unspecified, 3.4%; Asian or Native Hawaiian or Other Pacific Islander, 4.5%; Black, 10.7%; Hispanic, 11.9%; non-Hispanic, 88.1%; and White, 81.3%. Compared with respondents reporting chronic pain, those with surgical pain were more likely to be aged 65 years or older (34.7% vs 22.9%), female (58.0% vs 54.2%), White (85.0% vs 80.3%), non-Hispanic (90.3% vs 87.5%), not live in the western US (79.8% vs 74.7%), have 3 or more comorbidities (40.0% vs 26.6%), and experience extreme (8.7% vs 5.7%) pain interference (Table 1). This cohort was compared with all noninstitutionalized US adults between 2011 and 2019 (eTable 3 in the Supplement).

### Unadjusted Analyses

**Figure 1.**
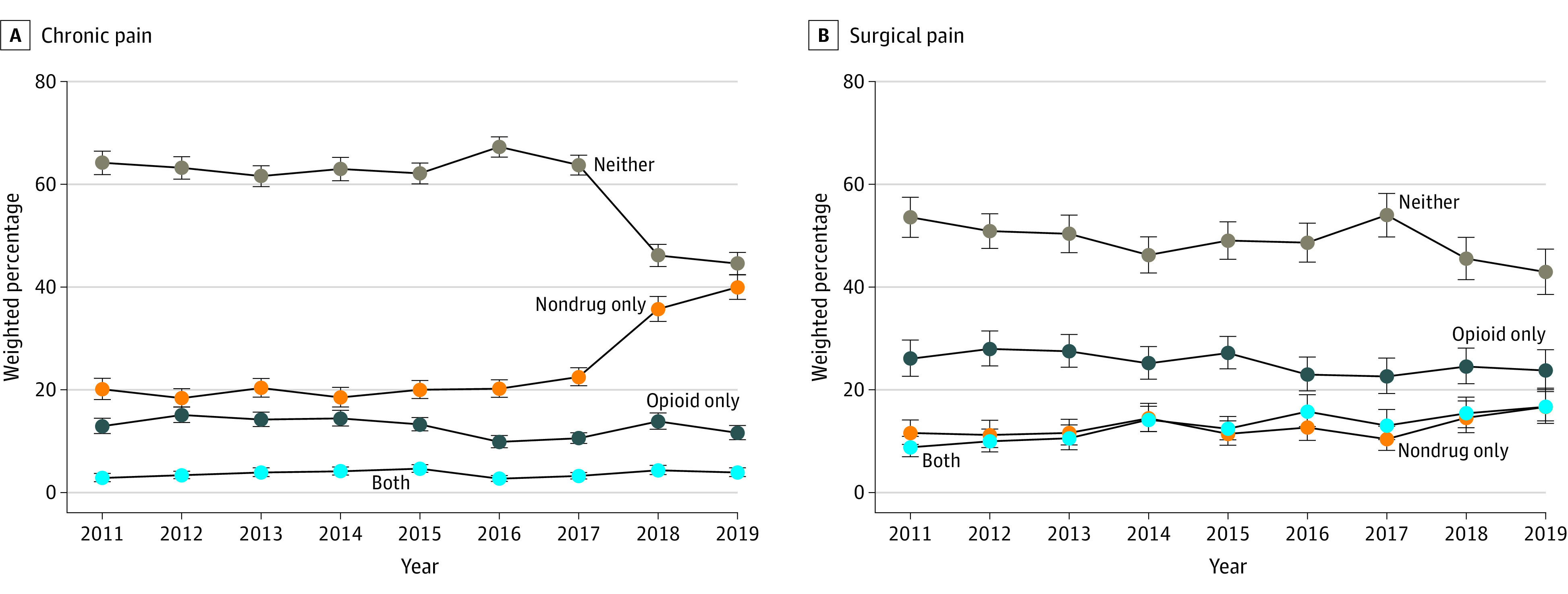
Trends in the Use of Mutually Exclusive Pain Treatments Trends from 2011 to 2019 in the mutually exclusive use of opioids, nondrug interventions, both treatments, or neither treatment in adults with chronic (A) or surgical (B) pain. Nondrug only is defined as any combination of acupuncture, chiropractic care, massage therapy, occupational therapy, or physical therapy. Weighted estimates are reported with 95% Clopper Pearson CIs as the error bars.

The annual weighted prevalence of mutually exclusive pain treatments is presented in Figure 1. Exclusive opioid use for chronic pain significantly decreased from 2014 (14.43%; 95% CI, 12.95%-16.01%) to 2017 (10.57%; 95% CI, 9.56%-11.63%), while nonpharmacologic treatments significantly increased from 2014 (18.50%; 95% CI, 16.64%-20.47%) to 2017 (22.50%; 95% CI, 20.78%-24.29%). By 2019, any opioid use decreased to 15.52% (95% CI, 13.99%-17.14%), while any nonpharmacologic use increased to 43.84% (95% CI, 41.44%-46.27%). Nonpharmacologic interventions never exceeded opioid use in the surgical cohort.

**Figure 2.**
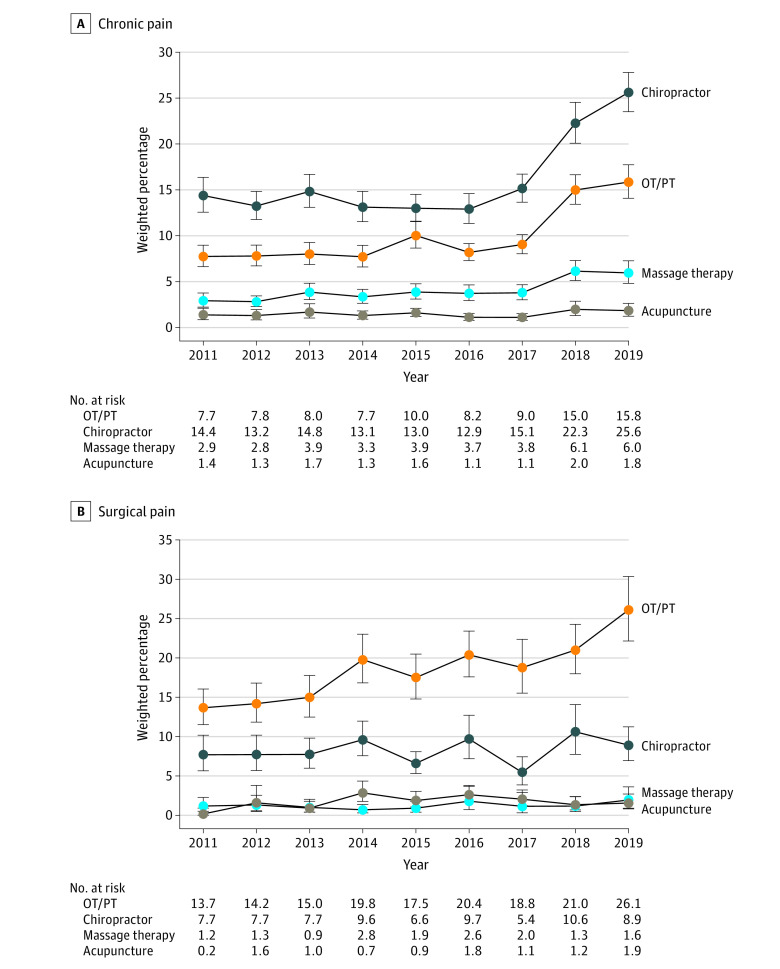
Trends in the Use of Any Nonpharmacologic Pain Treatment Trends from 2011 to 2019 in the use of any nonpharmacologic pain treatment including acupuncture, chiropractic care, massage therapy, occupational therapy and/or physical therapy (OT/PT) treatment in adults with chronic (A) or surgical (B) pain. Weighted estimates are reported with 95% Clopper Pearson CIs as the error bars.

The weighted prevalence of using any acupuncture, chiropractor, massage, and OT/PT intervention between 2011 and 2019 is reported in Figure 2. Between 2011 and 2016, OT represented 2.38% (95% CI, 1.54%-3.51%) of the OT/PT treatment group in the chronic pain cohort and 3.84% (95% CI, 2.44%-5.71%) in the surgical cohort. The use of chiropractic care continued to increase through 2019 (chronic pain, 25.6%) and (surgical pain, 8.9%).

Some respondents used other pharmacologic treatments known to reduce pain. Nonsteroidal anti-inflammatory drugs were used by 23.14% (95% CI, 20.26%-26.23%), and gabapentinoids (pregabalin and gabapentin) were used by 9.32% (95% CI, 7.52%-11.39%) of the neither treatment group with chronic pain in 2019. Use of selective serotonin reuptake inhibitors and serotonin-norepinephrine reuptake inhibitors was rare (<3%) and remained stable between 2011 and 2019 (eTable 4 in the Supplement).

### Adjusted Analyses

Adjusted logistic regression analyses for the chronic (Table 2) and surgical (Table 3) pain cohorts in each calendar year showed an association with pain treatment type after adjusting for demographic characteristics, socioeconomic status, health conditions, and pain interference severity. Results from the multivariable multinomial logit model sensitivity analyses stayed the same and noted that use of opioids alone became less likely than use of nonpharmacologic or both treatments in recent years (eTables 5 and 6 in the Supplement).

**Table 2.** Weighted Multivariable Analysis of Odds of Health Service Use Among Cancer-Free Adults With Chronic Pain—MEPS, 2011-2019

Characteristic	aOR (95% CI)			
	Opioids only (unweighted: n = 5159; weighted: n = 54 068 406)	Nondrug only (unweighted: n = 7119; weighted: n = 97 672 035)	Both (unweighted: n = 1237; weighted: n = 15 502 748)^a^	Neither treatment (unweighted: n = 23 262; weighted: n = 255 570 310)^b^
Year of pain treatment				
2011	1 [Reference]	1 [Reference]	1 [Reference]	1 [Reference]
2012	1.25 (1.06-1.47)	0.86 (0.73-1.01)	1.17 (0.85-1.61)	0.98 (0.87-1.09)
2013	1.16 (0.97-1.38)	1.01 (0.86-1.19)	1.48 (1.02-2.14)	0.86 (0.76-0.98)
2014	1.22 (1.01-1.47)	0.89 (0.74-1.06)	1.63 (1.19-2.23)	0.90 (0.78-1.04)
2015	1.11 (0.93-1.32)	0.94 (0.80-1.12)	1.70 (1.26-2.29)	0.90 (0.79-1.03)
2016	0.80 (0.66-0.96)	0.94 (0.80-1.10)	1.01 (0.72-1.41)	1.12 (0.99-1.27)
2017	0.92 (0.77-1.10)	1.07 (0.90-1.26)	1.28 (0.92-1.77)	0.93 (0.83-1.06)
2018	1.19 (0.98-1.45)	2.23 (1.89-2.64)	1.60 (1.13-2.27)	0.46 (0.40-0.52)
2019	0.94 (0.78-1.14)	2.72 (2.30-3.21)	1.43 (1.00-2.04)	0.43 (0.37-0.49)
Linear trend test of year				
*F*_1,396_	9.47	283.67	0.13	177.72
*P* value	<.002	<.001	.72	<.001
Pain interference				
Not at all	1 [Reference]	1 [Reference]	1 [Reference]	1 [Reference]
A little bit	1.43 (1.26-1.62)	1.05 (0.97-1.14)	1.94 (1.55-2.43)	0.83 (0.77-0.90)
Moderately	2.57 (2.23-2.95)	0.90 (0.81-1.01)	3.29 (2.60-4.15)	0.65 (0.60-0.71)
Quite a bit	4.20 (3.72-4.75)	0.70 (0.62-0.80)	5.33 (4.16-6.82)	0.44 (0.40-0.48)
Extremely	7.19 (6.18-8.37)	0.43 (0.35-0.53)	6.62 (5.09-8.61)	0.28 (0.24-0.32)
Age, y				
18-44	1 [Reference]	1 [Reference]	1 [Reference]	1 [Reference]
45-64	1.04 (0.92-1.16)	0.85 (0.78-0.93)	0.93 (0.77-1.11)	1.10 (1.02-1.19)
≥65	0.73 (0.64-0.83)	0.98 (0.88-1.09)	0.86 (0.70-1.07)	1.28 (1.17-1.41)
Sex				
Male	1 [Reference]	1 [Reference]	1 [Reference]	1 [Reference]
Female	1.02 (0.93-1.12)	1.32 (1.23-1.42)	1.24 (1.07-1.45)	0.78 (0.73-0.83)
Race and ethnicity				
American Indian or Alaska Native or unspecified	1.10 (0.87-1.40)	0.71 (0.59-0.85)	0.74 (0.52-1.05)	1.24 (1.06-1.44)
Asian or Native Hawaiian or Other Pacific Islander	0.46 (0.35-0.59)	0.81 (0.71-0.94)	0.34 (0.22-0.53)	1.68 (1.47-1.92)
Black	0.95 (0.84-1.07)	0.50 (0.44-0.56)	0.76 (0.62-0.94)	1.59 (1.45-1.74)
White	1 [Reference]	1 [Reference]	1 [Reference]	1 [Reference]
Hispanic				
No	1 [Reference]	1 [Reference]	1 [Reference]	1 [Reference]
Yes	0.73 (0.64-0.83)	0.71 (0.63-0.80)	0.74 (0.60-0.91)	1.58 (1.43-1.74)
Educational level				
No degree	1 [Reference]	1 [Reference]	1 [Reference]	1 [Reference]
GED or high school diploma	0.91 (0.81-1.04)	1.53 (1.32-1.77)	1.27 (0.98-1.64)	0.89 (0.80-0.98)
Associate, tech, or vocational degree	0.79 (0.67-0.93)	1.84 (1.56-2.16)	1.46 (1.10-1.93)	0.82 (0.73-0.92)
Bachelor’s degree	0.55 (0.46-0.67)	2.30 (1.96-2.70)	1.74 (1.26-2.39)	0.70 (0.62-0.79)
Master’s or doctoral degree	0.72 (0.59-0.90)	2.44 (2.05-2.90)	1.42 (0.98-2.06)	0.63 (0.55-0.72)
Unknown	0.98 (0.64-1.50)	1.39 (0.79-2.47)	0.28 (0.09-0.86)	1.04 (0.70-1.55)
Family income as % of poverty line^c^				
Poor/negative	1 [Reference]	1 [Reference]	1 [Reference]	1 [Reference]
Near poor	0.97 (0.81-1.15)	1.28 (1.03-1.59)	1.28 (0.89-1.83)	0.89 (0.77-1.04)
Low income	0.99 (0.86-1.13)	1.31 (1.09-1.56)	1.60 (1.20-2.14)	0.87 (0.78-0.97)
Middle income	0.87 (0.76-0.99)	1.63 (1.40-1.90)	1.56 (1.22-2.01)	0.82 (0.75-0.91)
High income	0.63 (0.56-0.72)	2.10 (1.80-2.44)	1.74 (1.32-2.29)	0.73 (0.65-0.81)
Census region				
Northeast	1 [Reference]	1 [Reference]	1 [Reference]	1 [Reference]
Midwest	1.52 (1.30-1.77)	1.18 (1.02-1.37)	1.22 (0.95-1.57)	0.72 (0.63-0.83)
South	1.81 (1.56-2.11)	0.60 (0.52-0.68)	0.76 (0.59-0.99)	1.15 (1.02-1.30)
West	1.54 (1.30-1.81)	1.15 (1.01-1.31)	1.16 (0.91-1.49)	0.75 (0.66-0.86)
Insurance coverage				
None	1 [Reference]	1 [Reference]	1 [Reference]	1 [Reference]
Private	1.39 (1.18-1.63)	1.09 (0.92-1.28)	1.91 (1.35-2.70)	0.73 (0.64-0.84)
Public only	1.84 (1.58-2.14)	0.74 (0.62-0.90)	1.70 (1.15-2.53)	0.78 (0.68-0.89)
No. of comorbidities^d^				
0	1 [Reference]	1 [Reference]	1 [Reference]	1 [Reference]
1	1.42 (1.24-1.62)	0.89 (0.81-0.98)	1.87 (1.46-2.39)	0.94 (0.87-1.02)
2	1.76 (1.49-2.07)	0.80 (0.72-0.90)	2.11 (1.62-2.74)	0.90 (0.82-0.99)
≥3	2.15 (1.85-2.50)	0.68 (0.61-0.75)	2.40 (1.84-3.11)	0.84 (0.77-0.91)

Abbreviations: aOR, adjusted odds ratio; GED, general educational development; MEPS, Medical Expenditure Panel Survey–Household Component.

^a^ Defined by use of opioids and nonpharmacologic treatments.

^b^ Defined by use of neither opioids nor nonpharmacologic therapy. The rates of other pharmacologic treatments known to reduce pain that were used by those in the neither treatment group can be viewed in eTable 4 in the Supplement.

^c^ Family income determined as percentage of poverty line (lowest income/negative [<100%], near lowest income [100% to <125%], low income [125% to <200%], middle income [200% to <400%], and high income [≥400%]).

^d^ Self-reported comorbidities included hypertension, coronary heart disease, high cholesterol level, emphysema, bronchitis, diabetes, arthritis, asthma, or stroke.

**Table 3.** Weighted Multivariable Analysis of Odds of Health Service Use Among Cancer-Free Adults With Surgical Pain—MEPS, 2011-2019

Characteristic	aOR (95% CI)			
	Opioids only (unweighted: n = 2572; weighted: n = 29 843 454)	Nondrug only (unweighted: n = 1089; weighted: n = 14 726 529)	Both (unweighted: n = 1118; weighted: n = 14 897 891)^a^	Neither treatment (unweighted: n = 4864; weighted: n = 57 484 194)^b^
Year of pain treatment				
2011	1 [Reference]	1 [Reference]	1 [Reference]	1 [Reference]
2012	1.11 (0.85-1.44)	0.90 (0.64-1.27)	1.18 (0.85-1.63)	0.92 (0.73-1.14)
2013	1.14 (0.89-1.46)	1.01 (0.76-1.35)	1.32 (0.92-1.90)	0.82 (0.66-1.02)
2014	0.99 (0.79-1.26)	1.29 (0.95-1.76)	1.76 (1.27-2.44)	0.72 (0.58-0.88)
2015	1.15 (0.91-1.47)	0.89 (0.65-1.22)	1.56 (1.14-2.13)	0.80 (0.65-0.98)
2016	0.89 (0.71-1.12)	1.04 (0.76-1.44)	1.99 (1.41-2.80)	0.80 (0.65-0.99)
2017	0.92 (0.71-1.19)	0.83 (0.60-1.14)	1.67 (1.18-2.36)	0.93 (0.74-1.17)
2018	1.01 (0.78-1.30)	1.19 (0.85-1.66)	2.00 (1.44-2.77)	0.68 (0.54-0.86)
2019	0.94 (0.71-1.24)	1.53 (1.13-2.08)	2.32 (1.63-3.29)	0.59 (0.46-0.75)
Linear trend test of year				
*F*_1,396_	3.02	4.45	16.89	8.06
*P* value	.08	<.04	<.001	.005
Pain interference				
Not at all	1 [Reference]	1 [Reference]	1 [Reference]	1 [Reference]
A little bit	1.00 (0.84-1.19)	1.08 (0.88-1.32)	1.85 (1.47-2.33)	0.77 (0.67-0.88)
Moderately	1.27 (1.07-1.50)	1.30 (1.04-1.62)	2.34 (1.83-3.01)	0.53 (0.46-0.62)
Quite a bit	1.63 (1.38-1.93)	1.05 (0.84-1.32)	3.92 (3.04-5.06)	0.36 (0.31-0.42)
Extremely	1.80 (1.46-2.23)	0.87 (0.63-1.19)	4.24 (3.11-5.77)	0.34 (0.28-0.41)
Age, y				
18-44	1 [Reference]	1 [Reference]	1 [Reference]	1 [Reference]
45-64	0.94 (0.80-1.11)	1.21 (0.97-1.51)	1.05 (0.84-1.31)	0.94 (0.81-1.10)
≥65	0.52 (0.43-0.63)	1.45 (1.14-1.83)	1.00 (0.77-1.30)	1.36 (1.15-1.61)
Sex				
Male	1 [Reference]	1 [Reference]	1 [Reference]	1 [Reference]
Female	0.96 (0.85-1.09)	1.27 (1.08-1.49)	1.00 (0.85-1.17)	0.93 (0.84-1.03)
Race and ethnicity				
American Indian or Alaska Native or unspecified	1.40 (0.99-1.96)	0.90 (0.59-1.36)	0.86 (0.56-1.30)	0.82 (0.62-1.09)
Asian or Native Hawaiian or Other Pacific Islander	0.67 (0.48-0.94)	0.62 (0.39-0.98)	0.63 (0.38-1.07)	1.95 (1.43-2.66)
Black	1.05 (0.91-1.22)	0.66 (0.52-0.85)	0.85 (0.67-1.08)	1.16 (1.01-1.33)
White	1 [Reference]	1 [Reference]	1 [Reference]	1 [Reference]
Hispanic				
No	1 [Reference]	1 [Reference]	1 [Reference]	1 [Reference]
Yes	0.89 (0.75-1.05)	1.06 (0.85-1.32)	0.77 (0.58-1.00)	1.19 (1.03-1.39)
Educational level				
No degree	1 [Reference]	1 [Reference]	1 [Reference]	1 [Reference]
Associate, tech, or vocational degree	1.13 (0.92-1.39)	1.48 (1.06-2.07)	1.21 (0.88-1.67)	0.74 (0.60-0.90)
Bachelor’s degree	0.78 (0.63-0.97)	1.78 (1.32-2.40)	1.36 (0.99-1.88)	0.83 (0.67-1.03)
GED or high school diploma	1.06 (0.89-1.26)	1.14 (0.88-1.49)	1.36 (1.01-1.83)	0.82 (0.69-0.98)
Master’s or doctoral degree	0.71 (0.53-0.96)	2.05 (1.45-2.88)	1.57 (1.10-2.26)	0.73 (0.57-0.94)
Unknown	1.27 (0.57-2.84)	1.58 (0.80-3.13)	0.62 (0.16-2.35)	0.84 (0.43-1.65)
Family income as % of poverty line^c^				
Poor/negative	1 [Reference]	1 [Reference]	1 [Reference]	1 [Reference]
Near poor	0.92 (0.71-1.20)	1.30 (0.84-2.00)	0.80 (0.51-1.25)	1.11 (0.86-1.43)
Low income	0.89 (0.74-1.06)	1.27 (0.90-1.79)	0.94 (0.68-1.30)	1.11 (0.92-1.33)
Middle income	0.82 (0.68-0.98)	1.52 (1.12-2.08)	1.46 (1.10-1.93)	0.94 (0.78-1.12)
High income	0.71 (0.58-0.85)	2.02 (1.49-2.74)	1.79 (1.31-2.45)	0.81 (0.66-0.98)
Census region				
Northeast	1 [Reference]	1 [Reference]	1 [Reference]	1 [Reference]
Midwest	1.35 (1.09-1.66)	0.92 (0.74-1.13)	1.01 (0.79-1.30)	0.84 (0.72-0.99)
South	1.65 (1.34-2.05)	0.71 (0.57-0.89)	0.67 (0.52-0.87)	0.95 (0.80-1.14)
West	1.36 (1.07-1.72)	1.02 (0.80-1.30)	0.95 (0.74-1.22)	0.81 (0.68-0.97)
Insurance coverage				
None	1 [Reference]	1 [Reference]	1 [Reference]	1 [Reference]
Private	0.92 (0.71-1.19)	1.35 (0.84-2.16)	1.84 (1.05-3.23)	0.87 (0.69-1.09)
Public only	1.01 (0.78-1.31)	1.30 (0.80-2.13)	1.28 (0.73-2.23)	0.95 (0.75-1.20)
No. of comorbidities^e^				
0	1 [Reference]	1 [Reference]	1 [Reference]	1 [Reference]
1	0.91 (0.75-1.12)	1.12 (0.86-1.44)	1.19 (0.91-1.57)	0.95 (0.81-1.11)
2	0.91 (0.74-1.12)	1.03 (0.77-1.37)	1.29 (0.98-1.70)	0.94 (0.78-1.12)
≥3	1.02 (0.82-1.28)	0.89 (0.68-1.16)	0.94 (0.71-1.23)	1.08 (0.89-1.30)

Abbreviations: aOR, adjusted odds ratio; GED, general educational development; MEPS, Medical Expenditure Panel Survey–Household Component.

^a^ Defined by use of opioids and nonpharmacologic treatments.

^b^ Defined by use of neither opioids nor nonpharmacologic therapy. The rates of other pharmacologic treatments known to reduce pain that were used by those in the neither treatment group can be viewed in eTable 4 in the Supplement.

^c^ Family income determined as percentage of poverty line (lowest income/negative [<100%], near lowest income [100% to <125%], low income [125% to <200%], middle income [200% to <400%], and high income [≥400%]).

### Chronic Pain

There were statistically significant linear trends for the adjusted annual use of prescription opioids, nonpharmacologic treatments, and neither treatment (Table 2). Compared with 2011, the adjusted odds of exclusively using nonpharmacologic treatments was unchanged in 2016 (adjusted odds ratio [aOR], 0.94; 95% CI, 0.80-1.10) but increased significantly by 2019 (aOR, 2.72; 95% CI, 2.30-3.21). The odds of using neither treatment in 2016 was not significant (aOR, 1.12; 95% CI, 0.99-1.27), but a significant decrease was noted in 2019 (aOR, 0.43; 95% CI, 0.37-0.49) compared with 2011. These treatments varied by pain interference severity (eTable 7 in the Supplement).

### Surgical Pain

The annual use of nonpharmacologic treatments, both, and neither treatment significantly changed over time (Table 3). Exclusive use of nonpharmacologic treatments increased in 2019 (aOR, 1.53; 95% CI, 1.13-2.08) compared with 2011. The odds of using both treatments increased in 2016 (aOR, 1.99; 95% CI, 1.41-2.80) and 2019 (aOR, 2.32; 95% CI, 1.63-3.29), while the odds of using neither treatment significantly decreased in 2016 (aOR, 0.80; 95% CI, 0.65-0.99) and in 2019 (aOR, 0.59; 95% CI, 0.46-0.75) compared with 2011.

### Post Hoc Sensitivity Analysis

A sensitivity analysis included respondents who self-reported pain interference via the Veterans RAND 12 Item Health Survey regardless of their diagnosis because some adults have pain without an *ICD* code or health care use. This new cohort was stratified by surgical status and had fair to moderate agreement with our *ICD*-based cohorts (nonsurgical, κ = 0.35; surgical, κ = 0.69). This approach may be limited by recall bias or by basing inclusion on 4 weeks of pain.^43^ Findings indicate (1) similar trends for acupuncture, chiropractic, massage, and OT/PT; (2) a larger prevalence of neither treatment (eFigure 2 and eFigure 3 in the Supplement); and (3) a less notable decrease in the odds of using neither treatment (eTable 8 in the Supplement).

## Discussion

Among cancer-free adults with pain, the exclusive use of prescription opioids was highest before the 2014 Drug Enforcement Administration policy^28^ and the 2016 CDC practice^20^ changes, similar to previous studies.^22,23,44,45^ Former MEPS research noted that state policy reduced new opioid starts by 4.9%, and prescribing guidelines resulted in an 11.7% increase in opioid discontinuations from 2014 to 2017.^32^ These declining rates of opioid use depend on prescription strength. Thus, our trends in opioid access may appear flatter because we did not capture the granular decrease in low-dose opioids coinciding with the increasing prevalence of high-dose opioids.^23,46^

This study provides evidence of a large increase in exclusively using nonpharmacologic treatments in 2016 to 2019 among persons with chronic pain. It is possible that this increase in nonpharmacologic treatments was a successful response to the CDC 2016 guidelines to reduce opioid prescribing for chronic pain. Research shows that timely access to OT/PT reduces prolonged opioid use in Medicare enrollees.^25,47,48^ However, the 2020 COVID-19 precautions and outpatient clinic closures transitioned patients away from nonpharmacologic treatments and increased their reliance on prescription opioid treatment.^26^ Future studies are needed to track whether nonpharmacologic treatments will continue to grow beyond 2019.

Nonpharmacologic treatments surpassed opioid treatment only among persons with chronic, nonsurgical pain. Compared with the CDC chronic pain guidelines, postsurgical guidelines may be less developed.^49^ Bundled payment policies for total joint arthroplasty have also restricted the use of postsurgical rehabilitation services, such as OT/PT, to reduce costs.^50^ Patients may also be transitioning to nonopioid analgesics. Similar to 2013 to 2018 trends among Medicare enrollees, we found increasing trends in the use of opioid substitutes, such as gabapentinoids, between 2011 and 2019.^51^ This transition coincides with a growing awareness of an increased risk for falls and other complications when central nervous system–acting medications are concomitantly prescribed.^10^ In addition, a limited referral network may explain the limited use of nonpharmacologic treatments. Previous studies^18,19^ used protocolized pain interventions delivered via research assistants to maximize internal validity; however, patients and clinicians may not know who to consult for these treatments in the clinical setting. A single academic medical institute found occupational therapists and physical therapists were inappropriately consulted 15% of the time prior to a multidisciplinary education and communication initiative.^52^ Streamlined communication between prescribers and the workforce specializing in nonpharmacologic pain interventions could improve care access.^24^

The most prevalent nonpharmacologic clinicians were chiropractors and physical therapists. Chiropractic care increased from 6.9% in 1990^53^ to 8.4% in 2012.^54^ Our study shows that use of chiropractic care continued increasing through 2019 (chronic pain, 25.6%) and (surgical pain, 8.9%). Historically, the annual number of visits for back pain has been similar for chiropractors and physical therapists according to MEPS research on 1999-2008 trends.^55^ Although PT was more costly,^55^ it was preferred among persons with greater disability and worse health.^56^

The least prevalent treatments were acupuncture and massage therapy. Their prevalence remains unchanged since as early as 1990,^53,57^ likely due to poor coverage from private and public payers.^41,58^ Acupuncture has been found to reduce opioid consumption following surgery^59^ but was not reimbursed by Medicare for low back pain until 2020.^60^ A recent MEPS study found all-cause acupuncturist visits increased from 0.4% to 0.8% between 2010 and 2019, with 50% or more of expenses paid out of pocket.^61^ Fewer than 4% of respondents in our OT/PT group used OT, indicating a similar prevalence to acupuncture and massage therapy. This finding was surprising because 96% of commercial and Medicare insurers cover OT, while only 33% cover acupuncture and 2% cover therapeutic massage for back pain.^41^ The restrained use of acupuncture, massage, and OT highlights an opportunity to further expand nonpharmacologic treatments. A Medicaid study examined the benefits of expanding reimbursement for acupuncture, chiropractic care, massage, and OT/PT and found 49.7% of 1789 patients receiving long-term opioid treatment were no longer prescribed opioids after 18 months.^62^

Respondents with chronic pain and pain interference became more likely to use nonpharmacologic treatments in 2016. Occupational therapists and physical therapists regularly treat patients with high acuity.^56,63^ Multidisciplinary rehabilitation, exercise, psychological therapies, and acupuncture can reasonably be adapted to patients with varying degrees of pain and acuity. However, beginning in 2018, such patients became more likely to use neither treatment. Since the CDC 2016 practice guidelines were instituted, MEPS research has found an increasing prevalence of using no pain treatment^32^ and a greater annual decrease in opioid use among patients with more severe pain.^45^ These findings warrant study and may reflect barriers to safer alternatives for those with severe pain interference. Accessing regular outpatient services is expensive, difficult with functional limitations impeding community mobility, problematic due to disparities in care access, and may conflict with occupational demands.^33,64,65^

### Limitations

Our study has several limitations. First, the findings cannot be generalized to institutionalized populations, active-duty military personnel, foreign visitors, or individuals with cancer-related pain, which necessitates different opioid prescribing practices.^20^ Second, some respondents received other prescriptions known to reduce pain (eg, duloxetine). Pharmacologic opioid substitutes were not within the scope of this study because prior research identified trends in the use of gabapentinoids, selective serotonin reuptake inhibitors, and serotonin-norepinephrine reuptake inhibitors for pain treatment.^51^ Use of gabapentinoids increased in prevalence over the study period but remained uncommon (<10%). Therefore, it is less likely that these medications were associated with the increases in nonpharmacologic treatments between 2016 and 2019. Similarly, updates to the MEPS survey in 2018 may capture more health service use.^66^ Our results note that nonpharmacologic treatments began increasing before this change and continued increasing through 2019. Third, beginning in 2017, MEPS collapsed OT and PT into a single category, which may increase the risk for measurement error. The low prevalence and myriad combinations of acupuncture, chiropractic, massage, and OT/PT led us to collapse nonpharmacologic treatments into a single category for multivariable analyses. Fourth, to increase the validity when identifying chronic pain, we used *ICD* codes from the Agency for Healthcare Research and Quality.^31,32,67^ However, some patients with pain might not receive health care, and *ICD* codes may miss this group of patients. Future MEPS studies could include respondents with consecutive rounds of pain responses to address this concern.^68^

## Conclusions

Between 2011 and 2019, the use of nonpharmacologic treatments increased while neither the use of opioids nor nonpharmacologic therapy decreased. The most common nonpharmacologic treatments were chiropractic care and PT, which surpassed opioid use for chronic pain. Greater pain interference increased the odds of using neither treatment for chronic pain. Our study holds broad clinical and policy relevance, including expanding the reimbursement for nonpharmacologic health care professionals and equalizing direct access—without a physician referral—between these professionals in some circumstances. Administrators and health care professionals may benefit from education on the effectiveness of nonpharmacologic treatments and which licensed professionals can be consulted to deliver such treatments.

## Notes

Supplement. **eFigure 1.** Cohort Flow Diagram **eTable 1.** Chronic Pain *ICD-9* and *ICD-10* Codes **eTable 2.** *ICD-9* and *ICD-10* Codes Used as Indication for Opioid Use **eTable 3.** Demographic Characteristics of Study Population and Target Population 2011-2019 MEPS Study Population **eTable 4.** Weighted Prevalence of Using Any Other Pharmacologic Pain Treatments Reported by the Mutually Exclusive Group Who Used “Neither Treatment” and Nonpharmacologic Treatments **eTable 5.** Weighted and Adjusted Multinomial Association Between Calendar Year and Mutually Exclusive Pain Treatment: Chronic Pain **eTable 6.** Weighted and Adjusted Multinomial Association Between Calendar Year and Mutually Exclusive Pain Treatment: Surgical Pain **eTable 7.** Weighted Multivariable Adjusted Odds for Annual Health Service Utilization Based on the Severity of Pain Interference Among Cancer-Free Adults – MEPS, 2011-2019 **eFigure 2.** Trends in the Use of Mutually Exclusive Pain Treatments Among Cancer-Free Adults With VR-12 Pain **eFigure 3.** Trends in the Use of Any Nonpharmacologic Pain Treatment Among Cancer-Free Adults With VR-12 Pain **eTable 8.** Weighted Multivariable Logistic Regression Analysis of Odds of Health Service Utilization Among Cancer-Free Adults With VR-12 Pain – MEPS, 2011-2019
eReferences
