# Supplementary material for: Trends in the Use of Opioids vs Nonpharmacologic Treatments in Adults With Pain, 2011-2019
Source: JAMA Netw Open. 2022 Nov 7;5(11):e2240612. doi: 10.1001/jamanetworkopen.2022.40612 (PMC9641539; doi:10.1001/jamanetworkopen.2022.40612)
Supplement: Supplement. — eFigure 1. Cohort Flow Diagram eTable 1. Chronic Pain ICD-9 and ICD-10 Codes eTable 2. ICD-9 and ICD-10 Codes Used as Indication for Opioid Use eTable 3. Demographic Characteristics of Study Population and Target Population 2011-2019 MEPS Study Population eTable 4. Weighted Prevalence of Using Any Other Pharmacologic Pain Treatments Reported by the Mutually Exclusive Group Who Used “Neither Treatment” and Nonpharmacologic Treatments eTable 5. Weighted and Adjusted Multinomial Association Between Calendar Year and Mutually Exclusive Pain Treatment: Chronic Pain eTable 6. Weighted and Adjusted Multinomial Association Between Calendar Year and Mutually Exclusive Pain Treatment: Surgical Pain eTable 7. Weighted Multivariable Adjusted Odds for Annual Health Service Utilization Based on the Severity of Pain Interference Among Cancer-Free Adults – MEPS, 2011-2019 eFigure 2. Trends in the Use of Mutually Exclusive Pain Treatments Among Cancer-Free Adults With VR-12 Pain eFigure 3. Trends in the Use of Any Nonpharmacologic Pain Treatment Among Cancer-Free Adults With VR-12 Pain eTable 8. Weighted Multivariable Logistic Regression Analysis of Odds of Health Service Utilization Among Cancer-Free Adults With VR-12 Pain – MEPS, 2011-2019 eReferences [file jamanetwopen-e2240612-s001.pdf]

Supplementary Online Content

Pritchard KT, Baillargeon J, Lee WC, Raji MA, Kuo YF. Trends in the use of opioids vs nonpharmacologic treatments in adults with pain, 2011-2019. *JAMA Netw Open*. 2022;5(11):e2240612. doi:10.1001/jamanetworkopen.2022.40612

eFigure 1. Cohort Flow Diagram

eTable 1. Chronic Pain *ICD-9* and *ICD-10* Codes

eTable 2. *ICD-9* and *ICD-10* Codes Used as Indication for Opioid Use

eTable 3. Demographic Characteristics of Study Population and Target Population 2011-2019 MEPS Study Population

eTable 4. Weighted Prevalence of Using Any Other Pharmacologic Pain Treatments Reported by the Mutually Exclusive Group Who Used “Neither Treatment” and Nonpharmacologic Treatments

eTable 5. Weighted and Adjusted Multinomial Association Between Calendar Year and Mutually Exclusive Pain Treatment: Chronic Pain

eTable 6. Weighted and Adjusted Multinomial Association Between Calendar Year and Mutually Exclusive Pain Treatment: Surgical Pain

eTable 7. Weighted Multivariable Adjusted Odds for Annual Health Service Utilization Based on the Severity of Pain Interference Among Cancer-Free Adults – MEPS, 2011-2019

eFigure 2. Trends in the Use of Mutually Exclusive Pain Treatments Among Cancer-Free Adults With VR-12 Pain

eFigure 3. Trends in the Use of Any Nonpharmacologic Pain Treatment Among Cancer-Free Adults With VR-12 Pain

eTable 8. Weighted Multivariable Logistic Regression Analysis of Odds of Health Service Utilization Among Cancer-Free Adults With VR-12 Pain – MEPS, 2011-2019

eReferences

This supplementary material has been provided by the authors to give readers additional information about their work.

© 2022 Pritchard KT et al. *JAMA Network Open.*

eFigure 1. Cohort Flow Diagram

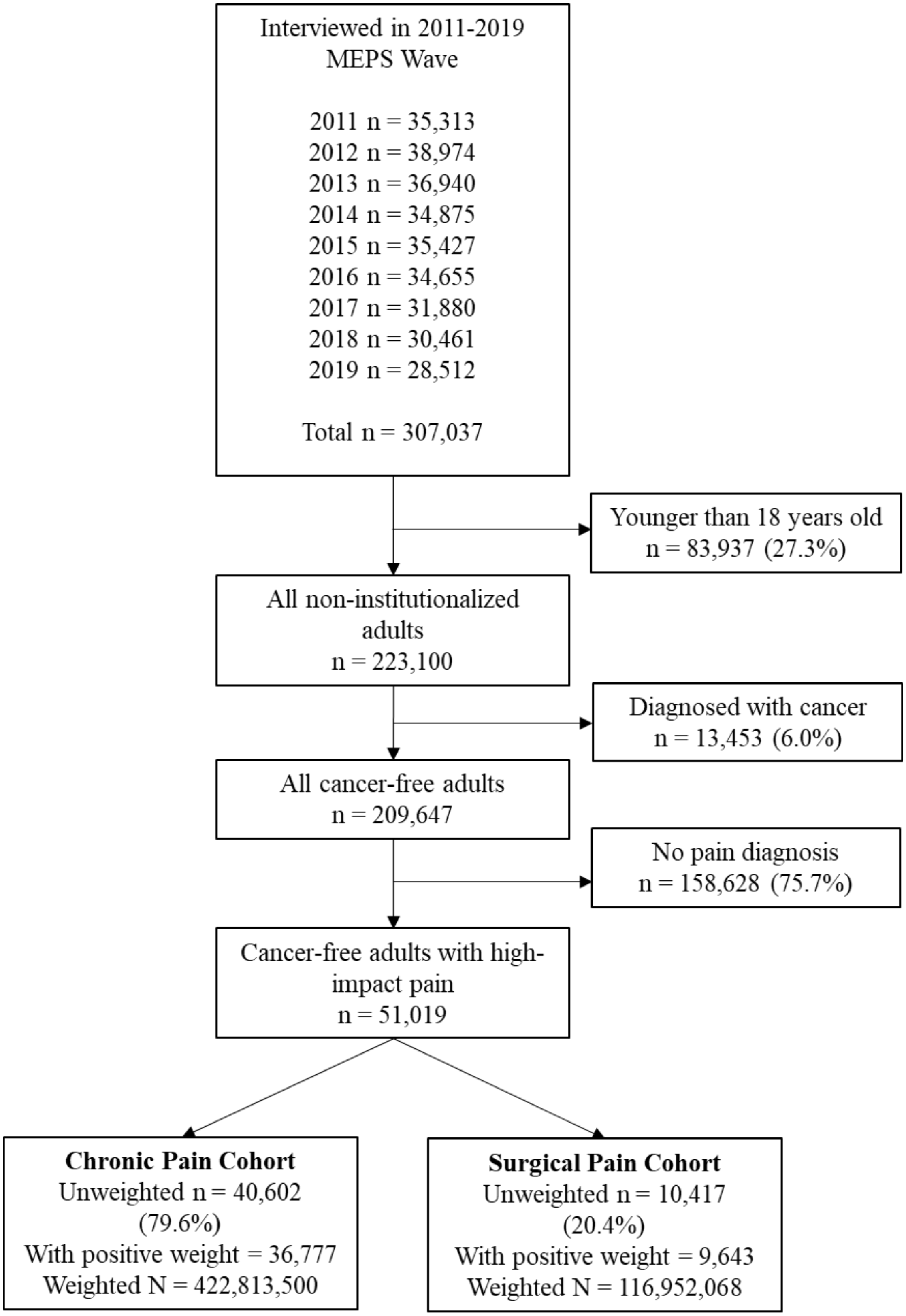

© 2022 Pritchard KT et al. *JAMA Network Open.*

eTable 1. Chronic Pain ICD-9 and ICD-10 Codes

ICD-9 code	ICD-9 code description	ICD-10 code	ICD-10 code description
307.8	Psychogenic pain, site unspecified	F45.41	Pain disorder exclusively related to psychological factors
307.89	Other pain disorders related to psychological factors	F45.42	Pain disorder with related psychological factors
338	Central pain syndrome	G89.0	Central pain syndrome
338.21	Chronic pain due to trauma	G89.21	Chronic pain due to trauma
338.22	Chronic post-thoracotomy pain	G89.22	Chronic post-thoracotomy pain
338.28	Other chronic postoperative pain	G89.28	Other chronic postprocedural pain
338.29	Other chronic pain	G89.29	Other chronic pain
338.4	Chronic pain syndrome	G89.4	Chronic pain syndrome
719.41	Pain in joint, shoulder region	M25.519	Pain in unspecified shoulder
719.45	Pain in joint, pelvic region and thigh	M25.559	Pain in unspecified hip
719.46	Pain in joint, lower leg	M25.569	Pain in unspecified knee
719.47	Pain in joint, ankle and foot	M25.579	Pain in unspecified ankle and joints of unspecified foot
719.49	Pain in joint, multiple sites	M25.50	Pain in unspecified joint
720	Ankylosing spondylitis	M45.9	Ankylosing spondylitis of unspecified sites in spine
720.2	Sacroiliitis, not elsewhere classified	M46.1	Sacroiliitis, not elsewhere classified
720.9	Unspecified inflammatory spondylopathy	M46.90	Unspecified inflammatory spondylopathy, site unspecified
721	Cervical spondylosis without myelopathy	M47.812	Spondylosis without myelopathy or radiculopathy, cervical region
721.1	Cervical spondylosis with myelopathy	M47.12	Other spondylosis with myelopathy, cervical region
721.2	Thoracic spondylosis without myelopathy	M47.814	Spondylosis without myelopathy or radiculopathy, thoracic region
721.3	Lumbosacral spondylosis without myelopathy	M47.817	Spondylosis without myelopathy or radiculopathy, lumbosacral region
721.41	Spondylosis with myelopathy, thoracic region	M47.14	Other spondylosis with myelopathy, thoracic region
721.42	Spondylosis with myelopathy, lumbar region	M47.16	Other spondylosis with myelopathy, lumbar region
721.6	Ankylosing vertebral hyperostosis	M48.10	Ankylosing hyperostosis [Forestier], site unspecified
721.8	Other allied disorders of spine	M48.9	Spondylopathy, unspecified
721.9	Spondylosis of unspecified site, without mention of myelopathy	M47.819	Spondylosis without myelopathy or radiculopathy, site unspecified
721.91	Spondylosis of unspecified site, with myelopathy	M47.10	Other spondylosis with myelopathy, site unspecified
722	Displacement of cervical intervertebral disc without myelopathy	M50.20	Other cervical disc displacement, unspecified cervical region
722.1	Displacement of lumbar intervertebral disc without myelopathy	M51.26	Other intervertebral disc displacement, lumbar region
722.1	Displacement of lumbar intervertebral disc without myelopathy	M51.27	Other intervertebral disc displacement, lumbosacral region

© 2022 Pritchard KT et al. *JAMA Network Open.*

ICD-9 code	ICD-9 code description	ICD-10 code	ICD-10 code description
722.11	Displacement of thoracic intervertebral disc without myelopathy	M51.24	Other intervertebral disc displacement, thoracic region
722.11	Displacement of thoracic intervertebral disc without myelopathy	M51.25	Other intervertebral disc displacement, thoracolumbar region
722.2	Displacement of intervertebral disc, site unspecified, without myelopathy	M51.9	Unspecified thoracic, thoracolumbar and lumbosacral intervertebral disc disorder
722.3	Schmorl's nodes, unspecified region	M51.9	Unspecified thoracic, thoracolumbar and lumbosacral intervertebral disc disorder
722.31	Schmorl's nodes, thoracic region	M51.44	Schmorl's nodes, thoracic region
722.31	Schmorl's nodes, thoracic region	M51.45	Schmorl's nodes, thoracolumbar region
722.32	Schmorl's nodes, lumbar region	M51.46	Schmorl's nodes, lumbar region
722.32	Schmorl's nodes, lumbar region	M51.47	Schmorl's nodes, lumbosacral region
722.39	Schmorl's nodes, other region	M51.9	Unspecified thoracic, thoracolumbar and lumbosacral intervertebral disc disorder
722.4	Degeneration of cervical intervertebral disc	M50.30	Other cervical disc degeneration, unspecified cervical region
722.51	Degeneration of thoracic or thoracolumbar intervertebral disc	M51.34	Other intervertebral disc degeneration, thoracic region
722.51	Degeneration of thoracic or thoracolumbar intervertebral disc	M51.35	Other intervertebral disc degeneration, thoracolumbar region
722.52	Degeneration of lumbar or lumbosacral intervertebral disc	M51.36	Other intervertebral disc degeneration, lumbar region
722.52	Degeneration of lumbar or lumbosacral intervertebral disc	M51.37	Other intervertebral disc degeneration, lumbosacral region
722.6	Degeneration of intervertebral disc, site unspecified	M51.34	Other intervertebral disc degeneration, thoracic region
722.6	Degeneration of intervertebral disc, site unspecified	M51.35	Other intervertebral disc degeneration, thoracolumbar region
722.6	Degeneration of intervertebral disc, site unspecified	M51.36	Other intervertebral disc degeneration, lumbar region
722.6	Degeneration of intervertebral disc, site unspecified	M51.37	Other intervertebral disc degeneration, lumbosacral region
722.7	Intervertebral disc disorder with myelopathy, unspecified region	M51.9	Unspecified thoracic, thoracolumbar and lumbosacral intervertebral disc disorder
722.71	Intervertebral disc disorder with myelopathy, cervical region	M50.00	Cervical disc disorder with myelopathy, unspecified cervical region
722.72	Intervertebral disc disorder with myelopathy, thoracic region	M51.04	Intervertebral disc disorders with myelopathy, thoracic region
722.72	Intervertebral disc disorder with myelopathy, thoracic region	M51.05	Intervertebral disc disorders with myelopathy, thoracolumbar region
722.73	Intervertebral disc disorder with myelopathy, lumbar region	M51.06	Intervertebral disc disorders with myelopathy, lumbar region
722.82	Postlaminectomy syndrome, thoracic region	M96.1	Postlaminectomy syndrome, not elsewhere classified
722.83	Postlaminectomy syndrome, lumbar region	M96.1	Postlaminectomy syndrome, not elsewhere classified
722.9	Other and unspecified disc disorder, unspecified region	M46.40	Discitis, unspecified, site unspecified

© 2022 Pritchard KT et al. *JAMA Network Open.*

ICD-9 code	ICD-9 code description	ICD-10 code	ICD-10 code description
722.9	Other and unspecified disc disorder, unspecified region	M51.9	Unspecified thoracic, thoracolumbar and lumbosacral intervertebral disc disorder
722.91	Other and unspecified disc disorder, cervical region	M50.80	Other cervical disc disorders, unspecified cervical region
722.91	Other and unspecified disc disorder, cervical region	M50.90	Cervical disc disorder, unspecified, unspecified cervical region
722.92	Other and unspecified disc disorder, thoracic region	M46.45	Discitis, unspecified, thoracolumbar region
722.92	Other and unspecified disc disorder, thoracic region	M51.84	Other intervertebral disc disorders, thoracic region
722.92	Other and unspecified disc disorder, thoracic region	M51.85	Other intervertebral disc disorders, thoracolumbar region
722.93	Other and unspecified disc disorder, lumbar region	M46.47	Discitis, unspecified, lumbosacral region
722.93	Other and unspecified disc disorder, lumbar region	M51.86	Other intervertebral disc disorders, lumbar region
722.93	Other and unspecified disc disorder, lumbar region	M51.87	Other intervertebral disc disorders, lumbosacral region
723	Spinal stenosis in cervical region	M48.02	Spinal stenosis, cervical region
723.1	Cervicalgia	M54.2	Cervicalgia
723.3	Cervicobrachial syndrome (diffuse)	M53.1	Cervicobrachial syndrome
723.4	Brachial neuritis or radiculitis NOS	M54.12	Radiculopathy, cervical region
723.4	Brachial neuritis or radiculitis NOS	M54.13	Radiculopathy, cervicothoracic region
723.5	Torticollis, unspecified	M43.6	Torticollis
723.6	Panniculitis specified as affecting neck	M54.02	Panniculitis affecting regions of neck and back, cervical region
723.7	Ossification of posterior longitudinal ligament in cervical region	M67.88	Other specified disorders of synovium and tendon, other site
723.8	Other syndromes affecting cervical region	M53.82	Other specified dorsopathies, cervical region
723.9	Unspecified musculoskeletal disorders and symptoms referable to neck	M53.82	Other specified dorsopathies, cervical region
724	Spinal stenosis, unspecified region	M48.00	Spinal stenosis, site unspecified
724.01	Spinal stenosis, thoracic region	M48.04	Spinal stenosis, thoracic region
724.02	Spinal stenosis, lumbar region, without neurogenic claudication	M48.06	Spinal stenosis, lumbar region
724.09	Spinal stenosis, other region	M48.08	Spinal stenosis, sacral and sacrococcygeal region
724.1	Pain in thoracic spine	M54.6	Pain in thoracic spine
724.2	Lumbago	M54.5	Low back pain
724.3	Sciatica	M54.30	Sciatica, unspecified side
724.4	Thoracic or lumbosacral neuritis or radiculitis, unspecified	M54.14	Radiculopathy, thoracic region
724.4	Thoracic or lumbosacral neuritis or radiculitis, unspecified	M54.15	Radiculopathy, thoracolumbar region
724.4	Thoracic or lumbosacral neuritis or radiculitis, unspecified	M54.16	Radiculopathy, lumbar region

© 2022 Pritchard KT et al. *JAMA Network Open.*

ICD-9 code	ICD-9 code description	ICD-10 code	ICD-10 code description
724.4	Thoracic or lumbosacral neuritis or radiculitis, unspecified	M54.17	Radiculopathy, lumbosacral region
724.5	Backache, unspecified	M54.89	Other dorsalgia
724.5	Backache, unspecified	M54.9	Dorsalgia, unspecified
724.6	Disorders of sacrum	M43.27	Fusion of spine, lumbosacral region
724.6	Disorders of sacrum	M43.28	Fusion of spine, sacral and sacrococcygeal region
724.6	Disorders of sacrum	M53.2X7	Spinal instabilities, lumbosacral region
724.6	Disorders of sacrum	M53.3	Sacrococcygeal disorders, not elsewhere classified
724.7	Unspecified disorder of coccyx	M53.3	Sacrococcygeal disorders, not elsewhere classified
724.79	Other disorders of coccyx	M53.3	Sacrococcygeal disorders, not elsewhere classified
724.8	Other symptoms referable to back	M54.08	Panniculitis affecting regions of neck and back, sacral and sacrococcygeal region
724.9	Other unspecified back disorders	M43.8X9	Other specified deforming dorsopathies, site unspecified
724.9	Other unspecified back disorders	M53.9	Dorsopathy, unspecified
729	Rheumatism, unspecified and fibrositis	M79.0	Rheumatism, unspecified
729.1	Myalgia and myositis, unspecified	M60.9	Myositis, unspecified
729.1	Myalgia and myositis, unspecified	M79.1	Myalgia
729.1	Myalgia and myositis, unspecified	M79.7	Fibromyalgia
729.2	Neuralgia, neuritis, and radiculitis, unspecified	M54.10	Radiculopathy, site unspecified
729.2	Neuralgia, neuritis, and radiculitis, unspecified	M79.2	Neuralgia and neuritis, unspecified
729.4	Fasciitis, unspecified	M72.9	Fibroblastic disorder, unspecified
729.5	Pain in limb	M79.609	Pain in unspecified limb

Note: The chronic pain cohort was defined using the methodology from the Weitzman Quality Institute,[1] which is used in Medical Expenditure Panel Survey research on chronic pain conducted by the Agency for Healthcare Research and Quality[2] and has been found to be reliable across the ICD-9 to ICD-10 transition in 2016.[3] This method has better validity than relying on pain scores alone. For example, the positive predictive value for the above ICD codes likely to represent chronic pain (94.9%) and for the ICD codes highly likely to represent chronic pain (88.9%) is better than the positive predictive value when using pain scores alone (75.0%).[1] Only the prescription opioids and nonpharmacologic treatments linked to these ICD codes were measured as chronic pain treatments.

© 2022 Pritchard KT et al. *JAMA Network Open.*

eTable 2. ICD-9 and ICD-10 Codes Used as Indication for Opioid Use

ICD-9 code	ICD-9 code description	ICD-10 code	ICD-10 code description
307.81	Tension headache	G44.209	Tension-type headache, unspecified, not intractable
339	Cluster headache syndrome, unspecified	G44.009	Cluster headache syndrome, unspecified, not intractable
784	Headache	G44.1	Vascular headache, not elsewhere classified
784	Headache	R51	Headache
720.1	Spinal enthesopathy	M46.00	Spinal enthesopathy, site unspecified
721	Cervical spondylosis without myelopathy	M47.812	Spondylosis without myelopathy or radiculopathy, cervical region
722	Displacement of cervical intervertebral disc without myelopathy	M50.20	Other cervical disc displacement, unspecified cervical region
723	Spinal stenosis in cervical region	M48.02	Spinal stenosis, cervical region
724	Spinal stenosis, unspecified region	M48.00	Spinal stenosis, site unspecified
756.1	Anomaly of spine, unspecified	Q76.49	Other congenital malformations of spine, not associated with scoliosis
725	Polymyalgia rheumatica	M35.3	Polymyalgia rheumatica
726	Adhesive capsulitis of shoulder	M75.00	Adhesive capsulitis of unspecified shoulder
727	Synovitis and tenosynovitis, unspecified	M65.9	Synovitis and tenosynovitis, unspecified
728.11	Progressive myositis ossificans	M61.10	Myositis ossificans progressiva, unspecified site
729	Rheumatism, unspecified and fibrositis	M79.0	Rheumatism, unspecified
781.99	Other symptoms involving nervous and musculoskeletal systems	R29.818	Other symptoms and signs involving the nervous system
781.99	Other symptoms involving nervous and musculoskeletal systems	R29.898	Other symptoms and signs involving the musculoskeletal system
781.99	Other symptoms involving nervous and musculoskeletal systems	R29.90	Unspecified symptoms and signs involving the nervous system
781.99	Other symptoms involving nervous and musculoskeletal systems	R29.91	Unspecified symptoms and signs involving the musculoskeletal system
830	Closed dislocation of jaw	S03.00XA	Dislocation of jaw, unspecified side, initial encounter
830	Closed dislocation of jaw	S03.01XA	Dislocation of jaw, right side, initial encounter
830	Closed dislocation of jaw	S03.02XA	Dislocation of jaw, left side, initial encounter
830	Closed dislocation of jaw	S03.03XA	Dislocation of jaw, bilateral, initial encounter
831	Closed dislocation of shoulder, unspecified	S43.006A	Unspecified dislocation of unspecified shoulder joint, initial encounter
832	Closed dislocation of elbow, unspecified	S53.006A	Unspecified dislocation of unspecified radial head, initial encounter

© 2022 Pritchard KT et al. *JAMA Network Open.*

832	Closed dislocation of elbow, unspecified	S53.106A	Unspecified dislocation of unspecified ulnohumeral joint, initial encounter
833	Closed dislocation of wrist, unspecified part	S63.006A	Unspecified dislocation of unspecified wrist and hand, initial encounter
834	Closed dislocation of finger, unspecified part	S63.106A	Unspecified dislocation of unspecified thumb, initial encounter
834	Closed dislocation of finger, unspecified part	S63.259A	Unspecified dislocation of unspecified finger, initial encounter
835	Closed dislocation of hip, unspecified site	S73.006A	Unspecified dislocation of unspecified hip, initial encounter
836	Tear of medial cartilage or meniscus of knee, current	S83.219A	Bucket-handle tear of medial meniscus, current injury, unspecified knee, initial encounter
836	Tear of medial cartilage or meniscus of knee, current	S83.249A	Other tear of medial meniscus, current injury, unspecified knee, initial encounter
837	Closed dislocation of ankle	S93.06XA	Dislocation of unspecified ankle joint, initial encounter
838	Closed dislocation of foot, unspecified	S93.306A	Unspecified dislocation of unspecified foot, initial encounter
839	Closed dislocation, cervical vertebra, unspecified	S13.101A	Dislocation of unspecified cervical vertebrae, initial encounter
840	Acromioclavicular (joint) (ligament) sprain	S43.50XA	Sprain of unspecified acromioclavicular joint, initial encounter
841	Radial collateral ligament sprain	S53.439A	Radial collateral ligament sprain of unspecified elbow, initial encounter
842	Sprain of wrist, unspecified site	S63.509A	Unspecified sprain of unspecified wrist, initial encounter
842	Sprain of wrist, unspecified site	S66.919A	Strain of unspecified muscle, fascia and tendon at wrist and hand level, unspecified hand, initial encounter
843	Iliofemoral (ligament) sprain	S73.119A	Iliofemoral ligament sprain of unspecified hip, initial encounter
844	Sprain of lateral collateral ligament of knee	S83.429A	Sprain of lateral collateral ligament of unspecified knee, initial encounter
845	Sprain of ankle, unspecified site	S93.409A	Sprain of unspecified ligament of unspecified ankle, initial encounter
845	Sprain of ankle, unspecified site	S96.919A	Strain of unspecified muscle and tendon at ankle and foot level, unspecified foot, initial encounter
846	Sprain of lumbosacral (joint) (ligament)	S33.8XXA	Sprain of other parts of lumbar spine and pelvis, initial encounter
847	Sprain of neck	S13.4XXA	Sprain of ligaments of cervical spine, initial encounter
847	Sprain of neck	S13.8XXA	Sprain of joints and ligaments of other parts of neck, initial encounter

© 2022 Pritchard KT et al. *JAMA Network Open.*

848	Sprain of septal cartilage of nose	S03.8XXA	Sprain of joints and ligaments of other parts of head, initial encounter
905.6	Late effect of dislocation	S03.00XS	Dislocation of jaw, unspecified side, sequela
905.6	Late effect of dislocation	S03.01XS	Dislocation of jaw, right side, sequela
905.6	Late effect of dislocation	S03.02XS	Dislocation of jaw, left side, sequela
905.6	Late effect of dislocation	S13.20XS	Dislocation of unspecified parts of neck, sequela
905.6	Late effect of dislocation	S23.20XS	Dislocation of unspecified part of thorax, sequela
905.6	Late effect of dislocation	S33.30XS	Dislocation of unspecified parts of lumbar spine and pelvis, sequela
905.6	Late effect of dislocation	S43.306S	Dislocation of unspecified parts of unspecified shoulder girdle, sequela
905.6	Late effect of dislocation	S63.006S	Unspecified dislocation of unspecified wrist and hand, sequela
905.6	Late effect of dislocation	S73.006S	Unspecified dislocation of unspecified hip, sequela
905.6	Late effect of dislocation	S83.006S	Unspecified dislocation of unspecified patella, sequela
905.6	Late effect of dislocation	S83.106S	Unspecified dislocation of unspecified knee, sequela
905.6	Late effect of dislocation	S93.06XS	Dislocation of unspecified ankle joint, sequela
905.6	Late effect of dislocation	S93.306S	Unspecified dislocation of unspecified foot, sequela
V43.60	Unspecified joint replacement	Z96.60	Presence of unspecified orthopedic joint implant
V48.3	Mechanical and motor problems with neck and trunk	R68.89	Other general symptoms and signs
V49.60	Unspecified level upper limb amputation status	Z89.209	Acquired absence of unspecified upper limb, unspecified level
800	Closed fracture of vault of skull without mention of intracranial injury, unspecified state of consciousness	S02.0XXA	Fracture of vault of skull, initial encounter for closed fracture
801	Closed fracture of base of skull without mention of intra cranial injury, unspecified state of consciousness	S02.101A	Fracture of base of skull, right side, initial encounter for closed fracture
801	Closed fracture of base of skull without mention of intra cranial injury, unspecified state of consciousness	S02.102A	Fracture of base of skull, left side, initial encounter for closed fracture
801	Closed fracture of base of skull without mention of intra cranial injury, unspecified state of consciousness	S02.109A	Fracture of base of skull, unspecified side, initial encounter for closed fracture
802	Closed fracture of nasal bones	S02.2XXA	Fracture of nasal bones, initial encounter for closed fracture
803	Other closed skull fracture without mention of intracranial injury, unspecified state of consciousness	S02.91XA	Unspecified fracture of skull, initial encounter for closed fracture

© 2022 Pritchard KT et al. *JAMA Network Open.*

804	Closed fractures involving skull or face with other bones, without mention of intracranial injury, unspecified state of consciousness	S02.91XA	Unspecified fracture of skull, initial encounter for closed fracture
805	Closed fracture of cervical vertebra, unspecified level	S12.9XXA	Fracture of neck, unspecified, initial encounter
806	Closed fracture of C1-C4 level with unspecified spinal cord injury	S14.101A	Unspecified injury at C1 level of cervical spinal cord, initial encounter
806	Closed fracture of C1-C4 level with unspecified spinal cord injury	S12.000A	Unspecified displaced fracture of first cervical vertebra, initial encounter for closed fracture
806	Closed fracture of C1-C4 level with unspecified spinal cord injury	S12.001A	Unspecified nondisplaced fracture of first cervical vertebra, initial encounter for closed fracture
806	Closed fracture of C1-C4 level with unspecified spinal cord injury	S14.102A	Unspecified injury at C2 level of cervical spinal cord, initial encounter
806	Closed fracture of C1-C4 level with unspecified spinal cord injury	S12.100A	Unspecified displaced fracture of second cervical vertebra, initial encounter for closed fracture
806	Closed fracture of C1-C4 level with unspecified spinal cord injury	S12.101A	Unspecified nondisplaced fracture of second cervical vertebra, initial encounter for closed fracture
806	Closed fracture of C1-C4 level with unspecified spinal cord injury	S14.103A	Unspecified injury at C3 level of cervical spinal cord, initial encounter
806	Closed fracture of C1-C4 level with unspecified spinal cord injury	S12.200A	Unspecified displaced fracture of third cervical vertebra, initial encounter for closed fracture
806	Closed fracture of C1-C4 level with unspecified spinal cord injury	S12.201A	Unspecified nondisplaced fracture of third cervical vertebra, initial encounter for closed fracture
806	Closed fracture of C1-C4 level with unspecified spinal cord injury	S14.104A	Unspecified injury at C4 level of cervical spinal cord, initial encounter
806	Closed fracture of C1-C4 level with unspecified spinal cord injury	S12.300A	Unspecified displaced fracture of fourth cervical vertebra, initial encounter for closed fracture
806	Closed fracture of C1-C4 level with unspecified spinal cord injury	S12.301A	Unspecified nondisplaced fracture of fourth cervical vertebra, initial encounter for closed fracture
807	Closed fracture of rib(s), unspecified	S22.39XA	Fracture of one rib, unspecified side, initial encounter for closed fracture
808	Closed fracture of acetabulum	S32.409A	Unspecified fracture of unspecified acetabulum, initial encounter for closed fracture
809	Fracture of bones of trunk, closed	S22.9XXA	Fracture of bony thorax, part unspecified, initial encounter for closed fracture
810	Closed fracture of clavicle, unspecified part	S42.009A	Fracture of unspecified part of unspecified clavicle, initial encounter for closed fracture

© 2022 Pritchard KT et al. *JAMA Network Open.*

811	Closed fracture of scapula, unspecified part	S42.109A	Fracture of unspecified part of scapula, unspecified shoulder, initial encounter for closed fracture
812	Closed fracture of unspecified part of upper end of humerus	S42.209A	Unspecified fracture of upper end of unspecified humerus, initial encounter for closed fracture
813	Closed fracture of upper end of forearm, unspecified	S52.90XA	Unspecified fracture of unspecified forearm, initial encounter for closed fracture
814	Closed fracture of carpal bone, unspecified	S62.109A	Fracture of unspecified carpal bone, unspecified wrist, initial encounter for closed fracture
815	Closed fracture of metacarpal bone(s), site unspecified	S62.309A	Unspecified fracture of unspecified metacarpal bone, initial encounter for closed fracture
816	Closed fracture of phalanx or phalanges of hand, unspecified	S62.509A	Fracture of unspecified phalanx of unspecified thumb, initial encounter for closed fracture
816	Closed fracture of phalanx or phalanges of hand, unspecified	S62.609A	Fracture of unspecified phalanx of unspecified finger, initial encounter for closed fracture
817	Multiple closed fractures of hand bones	S62.90XA	Unspecified fracture of unspecified wrist and hand, initial encounter for closed fracture
818	Ill-defined closed fractures of upper limb	S62.90XA	Unspecified fracture of unspecified wrist and hand, initial encounter for closed fracture
819	Multiple closed fractures involving both upper limbs, and upper limb with rib(s) and sternum	S42.91XA	Fracture of right shoulder girdle, part unspecified, initial encounter for closed fracture
819	Multiple closed fractures involving both upper limbs, and upper limb with rib(s) and sternum	S52.91XA	Unspecified fracture of right forearm, initial encounter for closed fracture
819	Multiple closed fractures involving both upper limbs, and upper limb with rib(s) and sternum	S42.92XA	Fracture of left shoulder girdle, part unspecified, initial encounter for closed fracture
819	Multiple closed fractures involving both upper limbs, and upper limb with rib(s) and sternum	S52.92XA	Unspecified fracture of left forearm, initial encounter for closed fracture
819	Multiple closed fractures involving both upper limbs, and upper limb with rib(s) and sternum	S42.90XA	Fracture of unspecified shoulder girdle, part unspecified, initial encounter for closed fracture
819	Multiple closed fractures involving both upper limbs, and upper limb with rib(s) and sternum	S52.90XA	Unspecified fracture of unspecified forearm, initial encounter for closed fracture
819	Multiple closed fractures involving both upper limbs, and upper limb with rib(s) and sternum	S22.20XA	Unspecified fracture of sternum, initial encounter for closed fracture
819	Multiple closed fractures involving both upper limbs, and upper limb with rib(s) and sternum	S22.49XA	Multiple fractures of ribs, unspecified side, initial encounter for closed fracture
820	Closed fracture of intracapsular section of neck of femur, unspecified	S72.019A	Unspecified intracapsular fracture of unspecified femur, initial encounter for closed fracture

© 2022 Pritchard KT et al. *JAMA Network Open.*

821	Closed fracture of unspecified part of femur	S72.90XA	Unspecified fracture of unspecified femur, initial encounter for closed fracture
822	Closed fracture of patella	S82.009A	Unspecified fracture of unspecified patella, initial encounter for closed fracture
823	Closed fracture of upper end of tibia alone	S82.109A	Unspecified fracture of upper end of unspecified tibia, initial encounter for closed fracture
824	Fracture of medial malleolus, closed	S82.53XA	Displaced fracture of medial malleolus of unspecified tibia, initial encounter for closed fracture
824	Fracture of medial malleolus, closed	S82.56XA	Nondisplaced fracture of medial malleolus of unspecified tibia, initial encounter for closed fracture
825	Fracture of calcaneus, closed	S92.009A	Unspecified fracture of unspecified calcaneus, initial encounter for closed fracture
825	Fracture of calcaneus, closed	S99.009A	Unspecified physeal fracture of unspecified calcaneus, initial encounter for closed fracture
825	Fracture of calcaneus, closed	S99.019A	Salter-Harris Type I physeal fracture of unspecified calcaneus, initial encounter for closed fracture
825	Fracture of calcaneus, closed	S99.029A	Salter-Harris Type II physeal fracture of unspecified calcaneus, initial encounter for closed fracture
825	Fracture of calcaneus, closed	S99.039A	Salter-Harris Type III physeal fracture of unspecified calcaneus, initial encounter for closed fracture
825	Fracture of calcaneus, closed	S99.049A	Salter-Harris Type IV physeal fracture of unspecified calcaneus, initial encounter for closed fracture
825	Fracture of calcaneus, closed	S99.099A	Other physeal fracture of unspecified calcaneus, initial encounter for closed fracture
826	Closed fracture of one or more phalanges of foot	S92.403A	Displaced unspecified fracture of unspecified great toe, initial encounter for closed fracture
826	Closed fracture of one or more phalanges of foot	S92.406A	Nondisplaced unspecified fracture of unspecified great toe, initial encounter for closed fracture
826	Closed fracture of one or more phalanges of foot	S92.503A	Displaced unspecified fracture of unspecified lesser toe(s), initial encounter for closed fracture
826	Closed fracture of one or more phalanges of foot	S92.506A	Nondisplaced unspecified fracture of unspecified lesser toe(s), initial encounter for closed fracture
827	Other, multiple and ill-defined fractures of lower limb, closed	S82.90XA	Unspecified fracture of unspecified lower leg, initial encounter for closed fracture
828	Closed multiple fractures involving both lower limbs, lower with upper limb, and lower limb(s) with rib(s) and sternum	S72.91XA	Unspecified fracture of right femur, initial encounter for closed fracture

© 2022 Pritchard KT et al. *JAMA Network Open.*

828	Closed multiple fractures involving both lower limbs, lower with upper limb, and lower limb(s) with rib(s) and sternum	S82.91XA	Unspecified fracture of right lower leg, initial encounter for closed fracture
828	Closed multiple fractures involving both lower limbs, lower with upper limb, and lower limb(s) with rib(s) and sternum	S72.92XA	Unspecified fracture of left femur, initial encounter for closed fracture
828	Closed multiple fractures involving both lower limbs, lower with upper limb, and lower limb(s) with rib(s) and sternum	S82.92XA	Unspecified fracture of left lower leg, initial encounter for closed fracture
828	Closed multiple fractures involving both lower limbs, lower with upper limb, and lower limb(s) with rib(s) and sternum	S72.90XA	Unspecified fracture of unspecified femur, initial encounter for closed fracture
828	Closed multiple fractures involving both lower limbs, lower with upper limb, and lower limb(s) with rib(s) and sternum	S82.90XA	Unspecified fracture of unspecified lower leg, initial encounter for closed fracture
828	Closed multiple fractures involving both lower limbs, lower with upper limb, and lower limb(s) with rib(s) and sternum	S42.90XA	Fracture of unspecified shoulder girdle, part unspecified, initial encounter for closed fracture
828	Closed multiple fractures involving both lower limbs, lower with upper limb, and lower limb(s) with rib(s) and sternum	S52.90XA	Unspecified fracture of unspecified forearm, initial encounter for closed fracture
828	Closed multiple fractures involving both lower limbs, lower with upper limb, and lower limb(s) with rib(s) and sternum	S72.90XA	Unspecified fracture of unspecified femur, initial encounter for closed fracture
828	Closed multiple fractures involving both lower limbs, lower with upper limb, and lower limb(s) with rib(s) and sternum	S82.90XA	Unspecified fracture of unspecified lower leg, initial encounter for closed fracture
828	Closed multiple fractures involving both lower limbs, lower with upper limb, and lower limb(s) with rib(s) and sternum	S22.49XA	Multiple fractures of ribs, unspecified side, initial encounter for closed fracture
828	Closed multiple fractures involving both lower limbs, lower with upper limb, and lower limb(s) with rib(s) and sternum	S22.20XA	Unspecified fracture of sternum, initial encounter for closed fracture
829	Fracture of unspecified bone, closed	T14.8	Other injury of unspecified body region
905	Late effect of fracture of skull and face bones	S02.610S	Fracture of condylar process of mandible, unspecified side, sequela
905	Late effect of fracture of skull and face bones	S02.632S	Fracture of coronoid process of left mandible, sequela
905	Late effect of fracture of skull and face bones	S02.631S	Fracture of coronoid process of right mandible, sequela
905	Late effect of fracture of skull and face bones	S02.630S	Fracture of coronoid process of mandible, unspecified side, sequela
905	Late effect of fracture of skull and face bones	S02.622S	Fracture of subcondylar process of left mandible, sequela
905	Late effect of fracture of skull and face bones	S02.621S	Fracture of subcondylar process of right mandible, sequela
905	Late effect of fracture of skull and face bones	S02.620S	Fracture of subcondylar process of mandible, unspecified side, sequela
905	Late effect of fracture of skull and face bones	S02.612S	Fracture of condylar process of left mandible, sequela

© 2022 Pritchard KT et al. *JAMA Network Open.*

905	Late effect of fracture of skull and face bones	S02.611S	Fracture of condylar process of right mandible, sequela
905	Late effect of fracture of skull and face bones	S02.640S	Fracture of ramus of mandible, unspecified side, sequela
905	Late effect of fracture of skull and face bones	S02.609S	Fracture of mandible, unspecified, sequela
905	Late effect of fracture of skull and face bones	S02.602S	Fracture of unspecified part of body of left mandible, sequela
905	Late effect of fracture of skull and face bones	S02.601S	Fracture of unspecified part of body of right mandible, sequela
905	Late effect of fracture of skull and face bones	S02.600S	Fracture of unspecified part of body of mandible, unspecified side, sequela
905	Late effect of fracture of skull and face bones	S02.5XXS	Fracture of tooth (traumatic), sequela
905	Late effect of fracture of skull and face bones	S02.42XS	Fracture of alveolus of maxilla, sequela
905	Late effect of fracture of skull and face bones	S02.413S	LeFort III fracture, sequela
905	Late effect of fracture of skull and face bones	S02.412S	LeFort II fracture, sequela
905	Late effect of fracture of skull and face bones	S02.641S	Fracture of ramus of right mandible, sequela
905	Late effect of fracture of skull and face bones	S02.642S	Fracture of ramus of left mandible, sequela
905	Late effect of fracture of skull and face bones	S02.650S	Fracture of angle of mandible, unspecified side, sequela
905	Late effect of fracture of skull and face bones	S02.651S	Fracture of angle of right mandible, sequela
905	Late effect of fracture of skull and face bones	S02.652S	Fracture of angle of left mandible, sequela
905	Late effect of fracture of skull and face bones	S02.66XS	Fracture of symphysis of mandible, sequela
905	Late effect of fracture of skull and face bones	S02.670S	Fracture of alveolus of mandible, unspecified side, sequela
905	Late effect of fracture of skull and face bones	S02.671S	Fracture of alveolus of right mandible, sequela
905	Late effect of fracture of skull and face bones	S02.672S	Fracture of alveolus of left mandible, sequela
905	Late effect of fracture of skull and face bones	S02.69XS	Fracture of mandible of other specified site, sequela
905	Late effect of fracture of skull and face bones	S02.80XS	Fracture of other specified skull and facial bones, unspecified side, sequela
905	Late effect of fracture of skull and face bones	S02.81XS	Fracture of other specified skull and facial bones, right side, sequela
905	Late effect of fracture of skull and face bones	S02.82XS	Fracture of other specified skull and facial bones, left side, sequela
905	Late effect of fracture of skull and face bones	S02.91XS	Unspecified fracture of skull, sequela
905	Late effect of fracture of skull and face bones	S02.92XS	Unspecified fracture of facial bones, sequela
905	Late effect of fracture of skull and face bones	S02.118S	Other fracture of occiput, unspecified side, sequela

© 2022 Pritchard KT et al. *JAMA Network Open.*

905	Late effect of fracture of skull and face bones	S02.11GS	Other fracture of occiput, right side, sequela
905	Late effect of fracture of skull and face bones	S02.11FS	Type III occipital condyle fracture, left side, sequela
905	Late effect of fracture of skull and face bones	S02.11ES	Type III occipital condyle fracture, right side, sequela
905	Late effect of fracture of skull and face bones	S02.11DS	Type II occipital condyle fracture, left side, sequela
905	Late effect of fracture of skull and face bones	S02.11CS	Type II occipital condyle fracture, right side, sequela
905	Late effect of fracture of skull and face bones	S02.11BS	Type I occipital condyle fracture, left side, sequela
905	Late effect of fracture of skull and face bones	S02.11AS	Type I occipital condyle fracture, right side, sequela
905	Late effect of fracture of skull and face bones	S02.119S	Unspecified fracture of occiput, sequela
905	Late effect of fracture of skull and face bones	S02.11HS	Other fracture of occiput, left side, sequela
905	Late effect of fracture of skull and face bones	S02.113S	Unspecified occipital condyle fracture, sequela
905	Late effect of fracture of skull and face bones	S02.112S	Type III occipital condyle fracture, unspecified side, sequela
905	Late effect of fracture of skull and face bones	S02.111S	Type II occipital condyle fracture, unspecified side, sequela
905	Late effect of fracture of skull and face bones	S02.110S	Type I occipital condyle fracture, unspecified side, sequela
905	Late effect of fracture of skull and face bones	S02.109S	Fracture of base of skull, unspecified side, sequela
905	Late effect of fracture of skull and face bones	S02.102S	Fracture of base of skull, left side, sequela
905	Late effect of fracture of skull and face bones	S02.101S	Fracture of base of skull, right side, sequela
905	Late effect of fracture of skull and face bones	S02.0XXS	Fracture of vault of skull, sequela
905	Late effect of fracture of skull and face bones	S02.19XS	Other fracture of base of skull, sequela
905	Late effect of fracture of skull and face bones	S02.2XXS	Fracture of nasal bones, sequela
905	Late effect of fracture of skull and face bones	S02.30XS	Fracture of orbital floor, unspecified side, sequela
905	Late effect of fracture of skull and face bones	S02.31XS	Fracture of orbital floor, right side, sequela
905	Late effect of fracture of skull and face bones	S02.32XS	Fracture of orbital floor, left side, sequela
905	Late effect of fracture of skull and face bones	S02.400S	Malar fracture, unspecified side, sequela
905	Late effect of fracture of skull and face bones	S02.401S	Maxillary fracture, unspecified side, sequela
905	Late effect of fracture of skull and face bones	S02.402S	Zygomatic fracture, unspecified side, sequela
905	Late effect of fracture of skull and face bones	S02.40AS	Malar fracture, right side, sequela
905	Late effect of fracture of skull and face bones	S02.40BS	Malar fracture, left side, sequela

© 2022 Pritchard KT et al. *JAMA Network Open.*

905	Late effect of fracture of skull and face bones	S02.40CS	Maxillary fracture, right side, sequela
905	Late effect of fracture of skull and face bones	S02.40DS	Maxillary fracture, left side, sequela
905	Late effect of fracture of skull and face bones	S02.40ES	Zygomatic fracture, right side, sequela
905	Late effect of fracture of skull and face bones	S02.40FS	Zygomatic fracture, left side, sequela
905	Late effect of fracture of skull and face bones	S02.411S	LeFort I fracture, sequela
V13.51	Personal history of pathologic fracture	Z87.311	Personal history of (healed) other pathological fracture
V66.4	Convalescence following treatment of fracture	Z51.89	Encounter for other specified aftercare
V67.4	Follow-up examination, following treatment of healed fracture	Z09	Encounter for follow-up examination after completed treatment for conditions other than malignant neoplasm
550	Inguinal hernia, with gangrene, unilateral or unspecified (not specified as recurrent)	K40.40	Unilateral inguinal hernia, with gangrene, not specified as recurrent
551	Femoral hernia with gangrene, unilateral or unspecified (not specified as recurrent)	K41.40	Unilateral femoral hernia, with gangrene, not specified as recurrent
552	Femoral hernia with obstruction, unilateral or unspecified (not specified as recurrent)	K41.30	Unilateral femoral hernia, with obstruction, without gangrene, not specified as recurrent
560.81	Intestinal or peritoneal adhesions with obstruction (postoperative) (postinfection)	K56.5	Intestinal adhesions [bands] with obstruction (postprocedural) (postinfection)
562.01	Diverticulitis of small intestine (without mention of hemorrhage)	K57.12	Diverticulitis of small intestine without perforation or abscess without bleeding
569.5	Abscess of intestine	K63.0	Abscess of intestine
574	Calculus of gallbladder with acute cholecystitis, without mention of obstruction	K80.00	Calculus of gallbladder with acute cholecystitis without obstruction
575	Acute cholecystitis	K81.0	Acute cholecystitis
577	Acute pancreatitis	K85.90	Acute pancreatitis without necrosis or infection, unspecified
577	Acute pancreatitis	K85.91	Acute pancreatitis with uninfected necrosis, unspecified
577	Acute pancreatitis	K85.92	Acute pancreatitis with infected necrosis, unspecified
592	Calculus of kidney	N20.0	Calculus of kidney
594	Calculus in diverticulum of bladder	N21.0	Calculus in bladder
596	Bladder neck obstruction	N32.0	Bladder-neck obstruction
788	Renal colic	N23	Unspecified renal colic
710	Systemic lupus erythematosus	M32.10	Systemic lupus erythematosus, organ or system involvement unspecified
711	Pyogenic arthritis, site unspecified	M00.00	Staphylococcal arthritis, unspecified joint

© 2022 Pritchard KT et al. *JAMA Network Open.*

711	Pyogenic arthritis, site unspecified	M00.10	Pneumococcal arthritis, unspecified joint
711	Pyogenic arthritis, site unspecified	M00.20	Other streptococcal arthritis, unspecified joint
711	Pyogenic arthritis, site unspecified	M00.80	Arthritis due to other bacteria, unspecified joint
711	Pyogenic arthritis, site unspecified	M00.9	Pyogenic arthritis, unspecified
713	Arthropathy associated with other endocrine and metabolic disorders	M14.80	Arthropathies in other specified diseases classified elsewhere, unspecified site
714	Rheumatoid arthritis	M06.9	Rheumatoid arthritis, unspecified
715	Osteoarthrosis, generalized, site unspecified	M15.0	Primary generalized (osteo)arthritis
715	Osteoarthrosis, generalized, site unspecified	M15.9	Polyosteoarthritis, unspecified
716	Kaschin-Beck disease, site unspecified	M12.10	Kaschin-Beck disease, unspecified site
717	Old bucket handle tear of medial meniscus	M23.205	Derangement of unspecified medial meniscus due to old tear or injury, unspecified knee
718	Articular cartilage disorder, site unspecified	M24.10	Other articular cartilage disorders, unspecified site
719	Effusion of joint, site unspecified	M25.40	Effusion, unspecified joint
720	Ankylosing spondylitis	M45.9	Ankylosing spondylitis of unspecified sites in spine
V13.4	Personal history of arthritis	Z87.39	Personal history of other diseases of the musculoskeletal system and connective tissue
860	Traumatic pneumothorax without mention of open wound into thorax	S27.0XXA	Traumatic pneumothorax, initial encounter
861	Unspecified injury of heart without mention of open wound into thorax	S26.10XA	Unspecified injury of heart without hemopericardium, initial encounter
861	Unspecified injury of heart without mention of open wound into thorax	S26.90XA	Unspecified injury of heart, unspecified with or without hemopericardium, initial encounter
861	Unspecified injury of heart without mention of open wound into thorax	S26.99XA	Other injury of heart, unspecified with or without hemopericardium, initial encounter
862	Injury to diaphragm, without mention of open wound into cavity	S27.809A	Unspecified injury of diaphragm, initial encounter
863	Injury to stomach, without mention of open wound into cavity	S36.30XA	Unspecified injury of stomach, initial encounter
864	Injury to liver without mention of open wound into cavity, unspecified injury	S36.119A	Unspecified injury of liver, initial encounter
865	Injury to spleen without mention of open wound into cavity, unspecified injury	S36.00XA	Unspecified injury of spleen, initial encounter
866	Injury to kidney without mention of open wound into cavity, unspecified injury	S37.009A	Unspecified injury of unspecified kidney, initial encounter
867	Injury to bladder and urethra, without mention of open wound into cavity	S37.20XA	Unspecified injury of bladder, initial encounter
867	Injury to bladder and urethra, without mention of open wound into cavity	S37.30XA	Unspecified injury of urethra, initial encounter

© 2022 Pritchard KT et al. *JAMA Network Open.*

868	Injury to other intra-abdominal organs without mention of open wound into cavity, unspecified intra-abdominal organ	S36.90XA	Unspecified injury of unspecified intra-abdominal organ, initial encounter
869	Internal injury to unspecified or ill-defined organs without mention of open wound into cavity	S36.90XA	Unspecified injury of unspecified intra-abdominal organ, initial encounter
869	Internal injury to unspecified or ill-defined organs without mention of open wound into cavity	S37.90XA	Unspecified injury of unspecified urinary and pelvic organ, initial encounter
870	Laceration of skin of eyelid and periocular area	S01.119A	Laceration without foreign body of unspecified eyelid and periocular area, initial encounter
870	Laceration of skin of eyelid and periocular area	S01.129A	Laceration with foreign body of unspecified eyelid and periocular area, initial encounter
870	Laceration of skin of eyelid and periocular area	S01.139A	Puncture wound without foreign body of unspecified eyelid and periocular area, initial encounter
870	Laceration of skin of eyelid and periocular area	S01.149A	Puncture wound with foreign body of unspecified eyelid and periocular area, initial encounter
870	Laceration of skin of eyelid and periocular area	S01.159A	Open bite of unspecified eyelid and periocular area, initial encounter
871	Ocular laceration without prolapse of intraocular tissue	S05.30XA	Ocular laceration without prolapse or loss of intraocular tissue, unspecified eye, initial encounter
872	Open wound of external ear, unspecified site, without mention of complication	S01.309A	Unspecified open wound of unspecified ear, initial encounter
873	Open wound of scalp, without mention of complication	S01.00XA	Unspecified open wound of scalp, initial encounter
874	Open wound of larynx with trachea, without mention of complication	S11.019A	Unspecified open wound of larynx, initial encounter
874	Open wound of larynx with trachea, without mention of complication	S11.029A	Unspecified open wound of trachea, initial encounter
875	Open wound of chest (wall), without mention of complication	S21.109A	Unspecified open wound of unspecified front wall of thorax without penetration into thoracic cavity, initial encounter
876	Open wound of back, without mention of complication	S21.209A	Unspecified open wound of unspecified back wall of thorax without penetration into thoracic cavity, initial encounter
876	Open wound of back, without mention of complication	S31.000A	Unspecified open wound of lower back and pelvis without penetration into retroperitoneum, initial encounter
877	Open wound of buttock, without mention of complication	S31.809A	Unspecified open wound of unspecified buttock, initial encounter
878	Open wound of penis, without mention of complication	S31.20XA	Unspecified open wound of penis, initial encounter
878	Open wound of penis, without mention of complication	S38.221A	Complete traumatic amputation of penis, initial encounter

© 2022 Pritchard KT et al. *JAMA Network Open.*

878	Open wound of penis, without mention of complication	S38.222A	Partial traumatic amputation of penis, initial encounter
879	Open wound of breast, without mention of complication	S21.009A	Unspecified open wound of unspecified breast, initial encounter
880	Open wound of shoulder region, without mention of complication	S41.009A	Unspecified open wound of unspecified shoulder, initial encounter
881	Open wound of forearm, without mention of complication	S51.809A	Unspecified open wound of unspecified forearm, initial encounter
882	Open wound of hand except finger(s) alone, without mention of complication	S61.409A	Unspecified open wound of unspecified hand, initial encounter
883	Open wound of finger(s), without mention of complication	S61.209A	Unspecified open wound of unspecified finger without damage to nail, initial encounter
884	Multiple and unspecified open wound of upper limb, without mention of complication	S41.009A	Unspecified open wound of unspecified shoulder, initial encounter
885	Traumatic amputation of thumb (complete)(partial), without mention of complication	S68.019A	Complete traumatic metacarpophalangeal amputation of unspecified thumb, initial encounter
885	Traumatic amputation of thumb (complete)(partial), without mention of complication	S68.029A	Partial traumatic metacarpophalangeal amputation of unspecified thumb, initial encounter
885	Traumatic amputation of thumb (complete)(partial), without mention of complication	S68.519A	Complete traumatic transphalangeal amputation of unspecified thumb, initial encounter
885	Traumatic amputation of thumb (complete)(partial), without mention of complication	S68.529A	Partial traumatic transphalangeal amputation of unspecified thumb, initial encounter
886	Traumatic amputation of other finger(s) (complete) (partial), without mention of complication	S68.119A	Complete traumatic metacarpophalangeal amputation of unspecified finger, initial encounter
886	Traumatic amputation of other finger(s) (complete) (partial), without mention of complication	S68.129A	Partial traumatic metacarpophalangeal amputation of unspecified finger, initial encounter
886	Traumatic amputation of other finger(s) (complete) (partial), without mention of complication	S68.619A	Complete traumatic transphalangeal amputation of unspecified finger, initial encounter
886	Traumatic amputation of other finger(s) (complete) (partial), without mention of complication	S68.629A	Partial traumatic transphalangeal amputation of unspecified finger, initial encounter
887	Traumatic amputation of arm and hand (complete) (partial), unilateral, below elbow, without mention of complication	S58.119A	Complete traumatic amputation at level between elbow and wrist, unspecified arm, initial encounter
887	Traumatic amputation of arm and hand (complete) (partial), unilateral, below elbow, without mention of complication	S58.129A	Partial traumatic amputation at level between elbow and wrist, unspecified arm, initial encounter

© 2022 Pritchard KT et al. *JAMA Network Open.*

887	Traumatic amputation of arm and hand (complete) (partial), unilateral, below elbow, without mention of complication	S58.919A	Complete traumatic amputation of unspecified forearm, level unspecified, initial encounter
887	Traumatic amputation of arm and hand (complete) (partial), unilateral, below elbow, without mention of complication	S58.929A	Partial traumatic amputation of unspecified forearm, level unspecified, initial encounter
887	Traumatic amputation of arm and hand (complete) (partial), unilateral, below elbow, without mention of complication	S68.419A	Complete traumatic amputation of unspecified hand at wrist level, initial encounter
887	Traumatic amputation of arm and hand (complete) (partial), unilateral, below elbow, without mention of complication	S68.429A	Partial traumatic amputation of unspecified hand at wrist level, initial encounter
887	Traumatic amputation of arm and hand (complete) (partial), unilateral, below elbow, without mention of complication	S68.719A	Complete traumatic transmetacarpal amputation of unspecified hand, initial encounter
887	Traumatic amputation of arm and hand (complete) (partial), unilateral, below elbow, without mention of complication	S68.729A	Partial traumatic transmetacarpal amputation of unspecified hand, initial encounter
890	Open wound of hip and thigh, without mention of complication	S71.009A	Unspecified open wound, unspecified hip, initial encounter
890	Open wound of hip and thigh, without mention of complication	S71.109A	Unspecified open wound, unspecified thigh, initial encounter
891	Open wound of knee, leg [except thigh], and ankle, without mention of complication	S81.009A	Unspecified open wound, unspecified knee, initial encounter
891	Open wound of knee, leg [except thigh], and ankle, without mention of complication	S81.809A	Unspecified open wound, unspecified lower leg, initial encounter
891	Open wound of knee, leg [except thigh], and ankle, without mention of complication	S91.009A	Unspecified open wound, unspecified ankle, initial encounter
892	Open wound of foot except toe(s) alone, without mention of complication	S91.309A	Unspecified open wound, unspecified foot, initial encounter
893	Open wound of toe(s), without mention of complication	S91.109A	Unspecified open wound of unspecified toe(s) without damage to nail, initial encounter
894	Multiple and unspecified open wound of lower limb, without mention of complication	S71.009A	Unspecified open wound, unspecified hip, initial encounter
895	Traumatic amputation of toe(s) (complete) (partial), without mention of complication	S98.119A	Complete traumatic amputation of unspecified great toe, initial encounter
895	Traumatic amputation of toe(s) (complete) (partial), without mention of complication	S98.129A	Partial traumatic amputation of unspecified great toe, initial encounter
895	Traumatic amputation of toe(s) (complete) (partial), without mention of complication	S98.139A	Complete traumatic amputation of one unspecified lesser toe, initial encounter
895	Traumatic amputation of toe(s) (complete) (partial), without mention of complication	S98.149A	Partial traumatic amputation of one unspecified lesser toe, initial encounter
895	Traumatic amputation of toe(s) (complete) (partial), without mention of complication	S98.219A	Complete traumatic amputation of two or more unspecified lesser toes, initial encounter

© 2022 Pritchard KT et al. *JAMA Network Open.*

895	Traumatic amputation of toe(s) (complete) (partial), without mention of complication	S98.229A	Partial traumatic amputation of two or more unspecified lesser toes, initial encounter
896	Traumatic amputation of foot (complete) (partial), unilateral, without mention of complication	S98.019A	Complete traumatic amputation of unspecified foot at ankle level, initial encounter
896	Traumatic amputation of foot (complete) (partial), unilateral, without mention of complication	S98.029A	Partial traumatic amputation of unspecified foot at ankle level, initial encounter
896	Traumatic amputation of foot (complete) (partial), unilateral, without mention of complication	S98.319A	Complete traumatic amputation of unspecified midfoot, initial encounter
896	Traumatic amputation of foot (complete) (partial), unilateral, without mention of complication	S98.329A	Partial traumatic amputation of unspecified midfoot, initial encounter
896	Traumatic amputation of foot (complete) (partial), unilateral, without mention of complication	S98.919A	Complete traumatic amputation of unspecified foot, level unspecified, initial encounter
896	Traumatic amputation of foot (complete) (partial), unilateral, without mention of complication	S98.929A	Partial traumatic amputation of unspecified foot, level unspecified, initial encounter
897	Traumatic amputation of leg(s) (complete) (partial), unilateral, below knee, without mention of complication	S88.119A	Complete traumatic amputation at level between knee and ankle, unspecified lower leg, initial encounter
897	Traumatic amputation of leg(s) (complete) (partial), unilateral, below knee, without mention of complication	S88.129A	Partial traumatic amputation at level between knee and ankle, unspecified lower leg, initial encounter
900	Injury to carotid artery, unspecified	S15.009A	Unspecified injury of unspecified carotid artery, initial encounter
901	Injury to thoracic aorta	S25.00XA	Unspecified injury of thoracic aorta, initial encounter
902	Injury to abdominal aorta	S35.00XA	Unspecified injury of abdominal aorta, initial encounter
903	Injury to axillary vessel(s), unspecified	S45.809A	Unspecified injury of other specified blood vessels at shoulder and upper arm level, unspecified arm, initial encounter
904	Injury to common femoral artery	S75.009A	Unspecified injury of femoral artery, unspecified leg, initial encounter
905.8	Late effect of tendon injury	M67.90	Unspecified disorder of synovium and tendon, unspecified site
906	Late effect of open wound of head, neck, and trunk	S01.90XS	Unspecified open wound of unspecified part of head, sequela
906	Late effect of open wound of head, neck, and trunk	S11.90XS	Unspecified open wound of unspecified part of neck, sequela
906	Late effect of open wound of head, neck, and trunk	S21.90XS	Unspecified open wound of unspecified part of thorax, sequela
906	Late effect of open wound of head, neck, and trunk	S31.000S	Unspecified open wound of lower back and pelvis without penetration into retroperitoneum, sequela

© 2022 Pritchard KT et al. *JAMA Network Open.*

906	Late effect of open wound of head, neck, and trunk	S31.109S	Unspecified open wound of abdominal wall, unspecified quadrant without penetration into peritoneal cavity, sequela
907.2	Late effect of spinal cord injury	S14.109S	Unspecified injury at unspecified level of cervical spinal cord, sequela
907.2	Late effect of spinal cord injury	S24.109S	Unspecified injury at unspecified level of thoracic spinal cord, sequela
907.2	Late effect of spinal cord injury	S34.109S	Unspecified injury to unspecified level of lumbar spinal cord, sequela
907.2	Late effect of spinal cord injury	S34.139S	Unspecified injury to sacral spinal cord, sequela
908	Late effect of internal injury to chest	S26.99XS	Other injury of heart, unspecified with or without hemopericardium, sequela
908	Late effect of internal injury to chest	S27.9XXS	Injury of unspecified intrathoracic organ, sequela
925.1	Crushing injury of face and scalp	S07.0XXA	Crushing injury of face, initial encounter
925.1	Crushing injury of face and scalp	S07.8XXA	Crushing injury of other parts of head, initial encounter
926	Crushing injury of external genitalia	S38.001A	Crushing injury of unspecified external genital organs, male, initial encounter
926	Crushing injury of external genitalia	S38.002A	Crushing injury of unspecified external genital organs, female, initial encounter
927	Crushing injury of shoulder region	S47.9XXA	Crushing injury of shoulder and upper arm, unspecified arm, initial encounter
928	Crushing injury of thigh	S77.10XA	Crushing injury of unspecified thigh, initial encounter
929	Crushing injury of multiple sites, not elsewhere classified	S77.20XA	Crushing injury of unspecified hip with thigh, initial encounter
940	Chemical burn of eyelids and periocular area	T26.50XA	Corrosion of unspecified eyelid and periocular area, initial encounter
941	Burn of unspecified degree of face and head, unspecified site	T20.00XA	Burn of unspecified degree of head, face, and neck, unspecified site, initial encounter
941	Burn of unspecified degree of face and head, unspecified site	T20.40XA	Corrosion of unspecified degree of head, face, and neck, unspecified site, initial encounter
942	Burn of unspecified degree of trunk, unspecified site	T21.00XA	Burn of unspecified degree of trunk, unspecified site, initial encounter
942	Burn of unspecified degree of trunk, unspecified site	T21.40XA	Corrosion of unspecified degree of trunk, unspecified site, initial encounter
943	Burn of unspecified degree of upper limb, except wrist and hand, unspecified site	T22.00XA	Burn of unspecified degree of shoulder and upper limb, except

© 2022 Pritchard KT et al. *JAMA Network Open.*

			wrist and hand, unspecified site, initial encounter
943	Burn of unspecified degree of upper limb, except wrist and hand, unspecified site	T22.40XA	Corrosion of unspecified degree of shoulder and upper limb, except wrist and hand, unspecified site, initial encounter
944	Burn of unspecified degree of hand, unspecified site	T23.009A	Burn of unspecified degree of unspecified hand, unspecified site, initial encounter
944	Burn of unspecified degree of hand, unspecified site	T23.409A	Corrosion of unspecified degree of unspecified hand, unspecified site, initial encounter
945	Burn of unspecified degree of lower limb [leg], unspecified site	T24.009A	Burn of unspecified degree of unspecified site of unspecified lower limb, except ankle and foot, initial encounter
945	Burn of unspecified degree of lower limb [leg], unspecified site	T24.409A	Corrosion of unspecified degree of unspecified site of unspecified lower limb, except ankle and foot, initial encounter
946	Burns of multiple specified sites, unspecified degree	T30.0	Burn of unspecified body region, unspecified degree
946	Burns of multiple specified sites, unspecified degree	T30.4	Corrosion of unspecified body region, unspecified degree
947	Burn of mouth and pharynx	T28.0XXA	Burn of mouth and pharynx, initial encounter
947	Burn of mouth and pharynx	T28.5XXA	Corrosion of mouth and pharynx, initial encounter
948	Burn [any degree] involving less than 10 percent of body surface with third degree burn, less than 10 percent or unspecified	T31.0	Burns involving less than 10% of body surface
948	Burn [any degree] involving less than 10 percent of body surface with third degree burn, less than 10 percent or unspecified	T32.0	Corrosions involving less than 10% of body surface
949	Burn of unspecified site, unspecified degree	T30.0	Burn of unspecified body region, unspecified degree
949	Burn of unspecified site, unspecified degree	T30.4	Corrosion of unspecified body region, unspecified degree
952	C1-C4 level with unspecified spinal cord injury	S14.101A	Unspecified injury at C1 level of cervical spinal cord, initial encounter
952	C1-C4 level with unspecified spinal cord injury	S14.102A	Unspecified injury at C2 level of cervical spinal cord, initial encounter
952	C1-C4 level with unspecified spinal cord injury	S14.103A	Unspecified injury at C3 level of cervical spinal cord, initial encounter
952	C1-C4 level with unspecified spinal cord injury	S14.104A	Unspecified injury at C4 level of cervical spinal cord, initial encounter
53.13	Postherpetic polyneuropathy	B02.23	Postherpetic polyneuropathy
72.72	Mumps polyneuropathy	B26.84	Mumps polyneuropathy
353	Brachial plexus lesions	G54.0	Brachial plexus disorders

© 2022 Pritchard KT et al. *JAMA Network Open.*

354	Carpal tunnel syndrome	G56.00	Carpal tunnel syndrome, unspecified upper limb
355	Lesion of sciatic nerve	G57.00	Lesion of sciatic nerve, unspecified lower limb
356	Hereditary peripheral neuropathy	G60.0	Hereditary motor and sensory neuropathy
357	Acute infective polyneuritis	G61.0	Guillain-Barré syndrome
377.33	Nutritional optic neuropathy	H46.2	Nutritional optic neuropathy
614	Acute salpingitis and oophoritis	N70.01	Acute salpingitis
614	Acute salpingitis and oophoritis	N70.02	Acute oophoritis
614	Acute salpingitis and oophoritis	N70.03	Acute salpingitis and oophoritis
615	Acute inflammatory diseases of uterus, except cervix	N71.0	Acute inflammatory disease of uterus
616.11	Vaginitis and vulvovaginitis in diseases classified elsewhere	N77.1	Vaginitis, vulvitis and vulvovaginitis in diseases classified elsewhere
617	Endometriosis of uterus	N80.0	Endometriosis of uterus
625.1	Vaginismus	N94.2	Vaginismus
626.3	Puberty bleeding	N92.2	Excessive menstruation at puberty
730	Acute osteomyelitis, site unspecified	M86.10	Other acute osteomyelitis, unspecified site
730	Acute osteomyelitis, site unspecified	M86.20	Subacute osteomyelitis, unspecified site
733	Osteoporosis, unspecified	M81.0	Age-related osteoporosis without current pathological fracture

Note: Due to a lack of procedural codes in the Medical Expenditure Panel Survey following 2016, ICD-9 and ICD-10 codes indicative of opioid use for painful conditions were used to identify the surgical pain cohort and surgical pain treatments. Respondents were included in the surgical cohort if they had a surgery according to the year's inpatient, emergency room, office-based, or outpatient files along with at least one of these ICD codes.[4,5] Notably, office-based surgeries are typically less invasive[6]; therefore, we used general anesthesia as a crude marker of invasiveness. Respondents with an office-based surgery and one of these ICD codes were included in the surgical cohort only if general anesthesia was used for their surgery. This aimed to reduce misclassification bias related to the definition of surgical pain. Only the prescription opioids and nonpharmacologic treatments linked to these ICD codes were measured as surgical pain treatments.

© 2022 Pritchard KT et al. *JAMA Network Open.*

eTable 3. Demographic Characteristics of Study Population and Target Population 2011-2019 MEPS Study Population.

	**Study Population: Included for Analysis** N=539,765,568; n=46,420			**Target Population: All Non-institutionalized Adults** N=539,765,568; n=193,288		
**Characteristic**	**Unweighted N**	**Weighted %**	**95 % CI**	**Unweighted N**	**Weighted %**	**95 % CI**
Year of Pain Treatment						
2011	4870	10.2	(9.8 - 10.7)	22515	10.7	(10.3 - 11.0)
2012	5534	10.3	(9.8 - 10.7)	25109	10.8	(10.5 - 11.1)
2013	5755	11.8	(11.4 - 12.3)	23077	10.9	(10.6 - 11.2)
2014	5629	12.7	(12.1 - 13.2)	21212	11.0	(10.7 - 11.4)
2015	5967	13.2	(12.6 - 13.7)	21633	11.2	(10.8 - 11.5)
2016	5863	12.6	(12.1 - 13.2)	21704	11.2	(10.9 - 11.6)
2017	5359	11.9	(11.4 - 12.5)	20615	11.3	(11.0 - 11.7)
2018	3938	8.9	(8.2 - 9.5)	19569	11.4	(10.7 - 12.1)
2019	3505	8.4	(7.8 - 8.9)	17854	11.4	(10.9 - 12.0)
Pain Interference						
Not At All	15614	35.5	(34.7 - 36.2)	112407	59.2	(58.7 - 59.7)
A Little Bit	12923	29.1	(28.5 - 29.6)	42144	22.3	(22.0 - 22.6)
Moderately	7219	15.2	(14.8 - 15.6)	17578	8.8	(8.6 - 9.0)
Quite a Bit	7258	13.9	(13.4 - 14.4)	14714	6.8	(6.6 - 7.0)
Extremely	3406	6.4	(6.0 - 6.7)	6445	2.9	(2.8 - 3.0)
Pain Intervention Type						
Neither Treatment [a]	28126	58.0	(57.2 - 58.8)	167890	85.5	(85.1 - 85.9)
Opioid Prescription Only	7731	15.5	(14.9 - 16.2)	11239	5.6	(5.4 - 5.9)
Nonpharmacologic Treatment Only	8208	20.8	(20.0 - 21.6)	11109	7.0	(6.8 - 7.3)
Both Opioid Prescription and Nonpharmacologic Treatment [b]	2355	5.6	(5.3 - 5.9)	3050	1.8	(1.7 - 1.9)
Age, years						
18-44	15158	32.9	(31.9 - 33.8)	90334	45.8	(45.1 - 46.5)
45-64	19510	41.7	(40.8 - 42.5)	65374	34.0	(33.6 - 34.5)
65+	11752	25.5	(24.6 - 26.3)	37580	20.2	(19.6 - 20.7)

© 2022 Pritchard KT et al. *JAMA Network Open.*

	Study Population: Included for Analysis<br>N=539,765,568; n=46,420			Target Population: All Non-institutionalized Adults<br>N=539,765,568; n=193,288		
**Characteristic**	**Unweighted N**	**Weighted %**	**95 % CI**	**Unweighted N**	**Weighted %**	**95 % CI**
Sex						
Female	26867	55.0	(54.4 - 55.7)	104612	51.8	(51.5 - 52.1)
Male	19553	45.0	(44.3 - 45.6)	88676	48.2	(47.9 - 48.5)
Race						
Asian/Native Hawaiian/Pacific Islander	2612	4.5	(4.0 - 5.1)	14125	6.1	(5.4 - 6.7)
Black	8592	10.7	(9.9 - 11.6)	36704	12.1	(11.2 - 13.0)
Other	1741	3.4	(2.9 - 3.8)	6284	3.1	(2.7 - 3.4)
White	33475	81.3	(80.2 - 82.5)	136175	78.8	(77.6 - 80.0)
Hispanic						
No	36885	88.1	(87.0 - 89.2)	142535	84.3	(83.0 - 85.5)
Yes	9535	11.9	(10.8 - 13.0)	50753	15.7	(14.5 - 17.0)
Education						
No Degree	8601	12.5	(11.8 - 13.1)	38498	13.4	(12.9 - 14.0)
GED or High School Diploma	19470	41.6	(40.8 - 42.5)	80940	41.6	(41.0 - 42.3)
Bachelor's Degree	7173	18.6	(17.9 - 19.3)	30334	19.3	(18.8 - 19.9)
Master's or Doctoral Degree	4314	11.8	(11.1 - 12.5)	16973	11.2	(10.7 - 11.7)
Associate, Tech, or Vocational Degree	6556	15.0	(14.4 - 15.6)	25034	13.8	(13.5 - 14.1)
Unknown	306	0.5	(0.4 - 0.6)	1509	0.6	(0.5 - 0.6)
Family income as % of Poverty Line						
Poor/Negative	8823	12.7	(12.1 - 13.4)	34552	11.6	(11.1 - 12.1)
Near Poor	2754	4.5	(4.2 - 4.8)	11077	4.1	(3.9 - 4.2)
Low Income	7118	12.7	(12.2 - 13.1)	30825	12.9	(12.6 - 13.2)
Middle Income	12806	27.5	(26.9 - 28.2)	56382	29.4	(28.9 - 29.9)
High Income	14919	42.6	(41.5 - 43.7)	60452	42.1	(41.1 - 43.0)
Census Region						
Northeast	7798	17.9	(16.6 - 19.2)	30677	17.9	(16.8 - 19.0)
Midwest	10179	22.9	(21.6 - 24.3)	37436	21.0	(20.0 - 22.1)

© 2022 Pritchard KT et al. *JAMA Network Open.*

	Study Population: Included for Analysis<br>N=539,765,568; n=46,420			Target Population: All Non-institutionalized Adults<br>N=539,765,568; n=193,288		
**Characteristic**	**Unweighted N**	**Weighted %**	**95 % CI**	**Unweighted N**	**Weighted %**	**95 % CI**
South	16413	35.0	(33.4 - 36.6)	73761	37.4	(36.1 - 38.8)
West	12030	24.2	(22.8 - 25.5)	51414	23.6	(22.5 - 24.7)
Insurance Coverage						
None	4268	7.0	(6.6 - 7.5)	29652	10.8	(10.3 - 11.3)
Private	27047	67.5	(66.5 - 68.5)	113323	68.6	(67.7 - 69.5)
Public Only	15105	25.5	(24.6 - 26.4)	50313	20.6	(20.0 - 21.3)
Number of Comorbidities						
0	12079	26.8	(26.0 - 27.5)	84073	42.9	(42.4 - 43.5)
1	10602	24.0	(23.4 - 24.7)	42428	22.7	(22.4 - 23.1)
2	9015	19.7	(19.1 - 20.3)	28344	15.2	(14.8 - 15.5)
3+	14724	29.5	(28.7 - 30.3)	38443	19.2	(18.7 - 19.6)

[a] The “Neither Treatment” group used neither opioids nor nonpharmacologic therapy. The rates of other pharmacologic treatments known to reduce pain that were used by those in the “Neither Treatment” group can be viewed in eTable 4.

[b] The “Both” treatments group was defined by using opioids and nonpharmacologic treatments.

Note: Pain interference was defined as how often pain interferes with work or daily life using the VR-12 (a little bit; moderately; quite a bit; extremely). Covariates included age (18-44; 44-64; and 65+ years); sex (female; male); race (White; Asian/Native Hawaiian/Pacific Islander; Black; Other including American Indian/Alaska Native/Unspecified); Hispanic ethnicity (yes; no); education (no degree; general educational development [GED] or high school diploma; associate/tech/or vocational; bachelor’s; master’s or doctoral; unknown); family income as percentage of poverty line (poor/negative [less than 100%]; near poor [100% to less than 125%]; low income [125% to less than 200%]; middle income [200% to less than 400%]; high income [greater than or equal to 400%]); census region (Northeast; Midwest; South; West); insurance type (none, private, or public only); number of self-reported comorbidities (hypertension, coronary heart disease, high cholesterol, emphysema, bronchitis, diabetes, arthritis, asthma, or stroke [0; 1; 2; ≥3]).

© 2022 Pritchard KT et al. *JAMA Network Open.*

eTable 4. Weighted Prevalence of Using Any Other Pharmacologic Pain Treatments Reported by the Mutually Exclusive Group Who Used (A.) "Neither Treatment" and (B.) Nonpharmacologic Treatments

A.			NSAIDs	SSRIs	SNRIs	Gabapentinoids
		**Year**	**% (95% CI)**	**% (95% CI)**	**% (95% CI)**	**% (95% CI)**
Neither Treatment [a] N=57,484,194 n=4,864	*Chronic Pain Cohort*	2011	13.60 (12.28-15.02)	0.51 (0.26-0.89)	0.65 (0.35-1.10)	3.00 (2.18-4.02)
		2015	14.19 (12.65-15.85)	0.26 (0.11-0.52)	0.47 (0.22-0.86)	4.03 (3.16-5.05)
		2019	23.14 (20.26-26.23)	0.94 (0.39-1.88)	2.67 (1.75-3.91)	9.32 (7.52-11.39)
		**Year**	**% (95% CI)**	**% (95% CI)**	**% (95% CI)**	**% (95% CI)**
	Surgical Cohort	2011	10.79 (7.60-14.72)	0.25 (0.02-1.04)	0.95 (0.19-2.78)	4.82 (2.75-7.78)
		2015	12.61 (9.52-16.26)	1.21 (0.33-3.07)	1.24 (0.38-2.96)	4.80 (2.89-7.45)
		2019	9.35 (5.81-14.06)	0.07 (0.00-1.19)	0.95 (0.55-2.78)	6.73 (4.24-10.06)

B.			NSAIDs	SSRIs	SNRIs	Gabapentinoids
		**Year**	**% (95% CI)**	**% (95% CI)**	**% (95% CI)**	**% (95% CI)**
Nonpharmacologic treatment N=14,726,529 n=1,089	*Chronic Pain Cohort*	2011	11.74 (8.59-15.55)	0.60 (0.10-1.92)	1.13 (0.39-2.52)	2.20 (1.15-3.77)
		2015	12.20 (9.69-15.10)	0.21 (0.02-0.83)	1.04 (0.23-2.93)	2.95 (1.82-4.52)
		2019	8.86 (6.97-11.05)	0.38 (0.10-1.01)	1.11 (0.47-2.22)	4.45 (3.15-6.08)
		**Year**	**% (95% CI)**	**% (95% CI)**	**% (95% CI)**	**% (95% CI)**
	Surgical Cohort	2011	12.59 (7.09-20.14)	3.73 (0.78-10.49)	2.27 (0.38-7.11)	1.93 (0.21-7.07)
		2015	16.36 (10.17-24.32)	N/A	1.14 (0.09-4.71)	9.31 (4.74-16.05)
		2019	13.62 (8.07-21.04)	N/A	0.34 (0.00-3.30)	7.93 (4.02-13.77)

[a] The "Neither Treatment" group used neither opioids nor nonpharmacologic therapy.

Note: Weighted percentages reported alongside 95% Clopper-Pearson (exact) 95% confidence intervals. Multum Lexicon database definitions for Nonsteroidal anti-inflammatory drugs (NSAIDs), Selective Serotonin Reuptake Inhibitors (SSRIs) Selective Serotonin Norepinephrine Reuptake Inhibitors (SNRIs), and Gabapentinoids were 61, 208, 308, and 347, respectively. Designation of "N/A" indicates that estimate was not possible due to small cell size after conditioning on cohort's pain type, year, and treatment type.

© 2022 Pritchard KT et al. *JAMA Network Open.*

eTable 5. Weighted and Adjusted Multinomial Association between Calendar Year and Mutually Exclusive pain Treatment: Chronic Pain (N=442,813,500; n=36,777)

**Opioids only (Reference)**	**Nondrug only**	**Both**	**Neither Treatment**
Year	aOR (95% CI)	aOR (95% CI)	aOR (95% CI)
2011	Reference	Reference	Reference
2012	0.72 (0.57-0.90)	0.97 (0.69-1.35)	0.81 (0.69-0.95)
2013	0.88 (0.69-1.11)	1.29 (0.88-1.90)	0.83 (0.70-0.98)
2014	0.75 (0.59-0.95)	1.35 (0.97-1.89)	0.80 (0.66-0.97)
2015	0.86 (0.68-1.09)	1.53 (1.12-2.07)	0.87 (0.73-1.04)
2016	1.17 (0.92-1.48)	1.21 (0.84-1.75)	1.27 (1.05-1.54)
2017	1.13 (0.89-1.45)	1.36 (0.97-1.91)	1.05 (0.88-1.25)
2018	1.56 (1.22-2.01)	1.43 (0.98-2.10)	0.63 (0.51-0.77)
2019	2.23 (1.74-2.85)	1.59 (1.09-2.32)	0.76 (0.62-0.93)

Note: Multivariable multinomial regression adjusted for sociodemographic factors (age, sex, race, ethnicity, census region), socioeconomic status (income, education, insurance type), number of comorbidities (hypertension, coronary heart disease, emphysema, chronic bronchitis, diabetes, arthritis, asthma, stroke), and severity of pain interference. Mutually exclusive outcomes for non-pharmacologic, both treatments, and neither treatment are in reference to those who used opioids alone.

Design-adjusted multiparameter Wald test for the effect of year on the multinomial outcomes controlling for all covariates: $F_{24,373}$ = 21.98 $p < .001$

Adjusted Odds Ratio (aOR); Confidence Interval (95% CI)

© 2022 Pritchard KT et al. *JAMA Network Open.*

eTable 6. Weighted and Adjusted Multinomial Association between Calendar Year and Mutually Exclusive Pain Treatment: Surgical Pain (N=116,952,068; n=9,643)

**Opioids only (Reference)**	**Nondrug only**	**Both**	**Neither Treatment**
Year	aOR (95% CI)	aOR (95% CI)	aOR (95% CI)
2011	Reference	Reference	Reference
2012	0.84 (0.56-1.26)	1.07 (0.73-1.57)	0.89 (0.67-1.17)
2013	0.92 (0.65-1.30)	1.17 (0.78-1.76)	0.82 (0.63-1.07)
2014	1.27 (0.88-1.83)	1.67 (1.15-2.42)	0.85 (0.66-1.08)
2015	0.82 (0.57-1.17)	1.33 (0.93-1.92)	0.80 (0.62-1.03)
2016	1.14 (0.79-1.66)	1.99 (1.40-2.84)	0.97 (0.76-1.25)
2017	0.91 (0.63-1.32)	1.67 (1.13-2.48)	1.02 (0.78-1.35)
2018	1.16 (0.79-1.71)	1.85 (1.27-2.68)	0.82 (0.62-1.07)
2019	1.54 (1.06-2.23)	2.23 (1.47-3.38)	0.79 (0.59-1.07)

Note: Multivariable multinomial regression adjusted for sociodemographic factors (age, sex, race, ethnicity, census region), socioeconomic status (income, education, insurance type), number of comorbidities (hypertension, coronary heart disease, emphysema, chronic bronchitis, diabetes, arthritis, asthma, stroke), and severity of pain interference. Mutually exclusive outcomes for non-pharmacologic, both treatments, and neither treatment are in reference to those who used opioids alone.

Design-adjusted multiparameter Wald test for the effect of year on the multinomial outcomes controlling for all covariates: $F_{24,373} = 3.29$ $p < .001$

Adjusted Odds Ratio (aOR); Confidence Interval (95% CI)

© 2022 Pritchard KT et al. *JAMA Network Open.*

eTable 7. Weighted Multivariable Adjusted Odds for Annual Health Service Utilization Based on the Severity of Pain Interference among Cancer-free Adults – MEPS, 2011-2019.

Pain Interference Severity (Reference = None)	Year	A Little Bit aOR (95% CI)	Moderate aOR (95% CI)	Quite A Bit aOR (95% CI)	Extremely aOR (95% CI)
**Nondrug Only:** Chronic Pain N=97,672,035 n=7,119	2011	Reference	Reference	Reference	Reference
	2012	1.05 (0.71-1.56)	1.16 (0.73-1.87)	0.73 (0.41-1.29)	0.94 (0.42-2.12)
	2013	1.17 (0.80-1.72)	1.20 (0.70-2.06)	1.02 (0.61-1.72)	0.77 (0.34-1.73)
	2014	0.93 (0.65-1.33)	0.86 (0.52-1.43)	0.82 (0.48-1.41)	1.17 (0.50-2.72)
	2015	1.03 (0.71-1.49)	1.22 (0.75-1.96)	1.43 (0.86-2.38)	1.07 (0.51-2.25)
	2016	1.38 (1.00-1.92)	1.63 (1.04-2.54)	1.85 (1.08-3.15)	1.57 (0.75-3.28)
	2017	0.89 (0.63-1.27)	1.03 (0.64-1.65)	1.31 (0.80-2.16)	1.23 (0.52-2.91)
	2018	0.65 (0.46-0.92)	0.60 (0.36-0.98)	0.47 (0.28-0.81)	0.31 (0.14-0.68)
	2019	0.86 (0.61-1.22)	0.78 (0.49-1.24)	0.70 (0.42-1.19)	0.73 (0.33-1.61)
**Neither Treatment [a]** Chronic Pain N=255,570,310 n=23,262	2011	Reference	Reference	Reference	Reference
	2012	0.91 (0.65-1.25)	0.71 (0.49-1.03)	0.77 (0.53-1.11)	0.84 (0.51-1.40)
	2013	0.90 (0.65-1.27)	0.75 (0.50-1.14)	0.98 (0.69-1.39)	0.90 (0.54-1.50)
	2014	1.00 (0.72-1.38)	0.69 (0.47-1.00)	0.67 (0.47-0.94)	0.56 (0.34-0.93)
	2015	1.04 (0.77-1.41)	0.78 (0.53-1.14)	0.70 (0.50-0.99)	0.97 (0.61-1.56)
	2016	0.76 (0.57-1.01)	0.69 (0.48-1.00)	0.64 (0.47-0.89)	1.06 (0.69-1.64)
	2017	1.11 (0.82-1.51)	0.98 (0.67-1.41)	0.99 (0.72-1.36)	1.12 (0.71-1.78)
	2018	1.45 (1.05-1.98)	1.49 (1.01-2.20)	2.28 (1.54-3.37)	3.26 (2.00-5.31)
	2019	1.10 (0.79-1.53)	1.25 (0.85-1.83)	1.73 (1.21-2.48)	2.82 (1.66-4.81)

[a] "Neither Treatment" group used neither opioids nor nonpharmacologic therapy. The rates of other pharmacologic treatments known to reduce pain that were used by those in the "Neither Treatment" group can be viewed in eTable 4.

Note: After adjusting for sociodemographic factors (age, sex, race, ethnicity, census region), socioeconomic status (income, education, insurance type), the number of comorbidities (hypertension, coronary heart disease, emphysema, chronic bronchitis, diabetes, arthritis, asthma, stroke), the annual use of pain treatments did not depend on the severity of pain interference for the following groups: Those exclusively using opioids (chronic pain, $p_{interaction} = 0.14$; surgical, $p_{interaction} = 0.15$), those with surgical pain exclusively using nonpharmacologic treatments ($p_{interaction} = 0.93$), those using both treatments (chronic pain, $p_{interaction} = 0.48$; surgical, $p_{interaction} = 0.41$), and those using neither treatment (surgical, $p_{interaction} = 0.62$). Among the chronic pain cohort, the annual use of nonpharmacologic treatments ($p_{interaction} < 0.001$) and neither treatment ($p_{interaction} < 0.001$) did vary based on the severity of pain interference. Therefore, interaction analyses were only reported above if the interaction was statistically significant.

Adjusted Odds Ratio (aOR); Confidence Interval (95% CI)

© 2022 Pritchard KT et al. *JAMA Network Open.*

eFigure 2. Trends in the use of mutually exclusive pain treatments among Cancer-free Adults with VR-12 Pain.

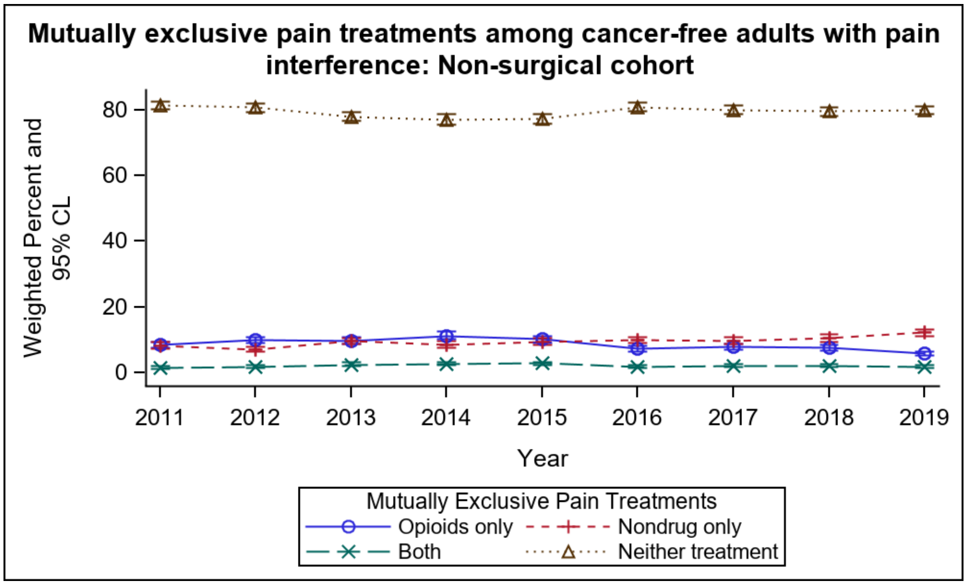

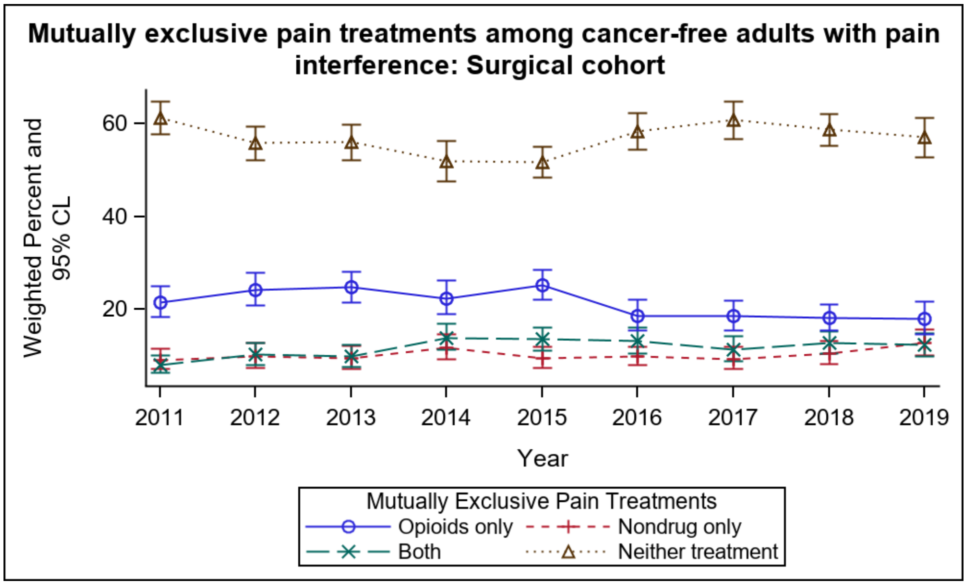

Note: 2011-2019 trends in the mutually exclusive utilization of opioids, non-drug, both, or neither treatment. Non-drug only is defined as any combination of acupuncture, chiropractic care, massage therapy, occupational therapy, or physical therapy. Weighted estimates are reported with 95% Clopper Pearson confidence intervals.

© 2022 Pritchard KT et al. *JAMA Network Open.*

eFigure 3. Trends in the use of any nonpharmacologic pain treatment among Cancer-free Adults with VR-12 Pain.

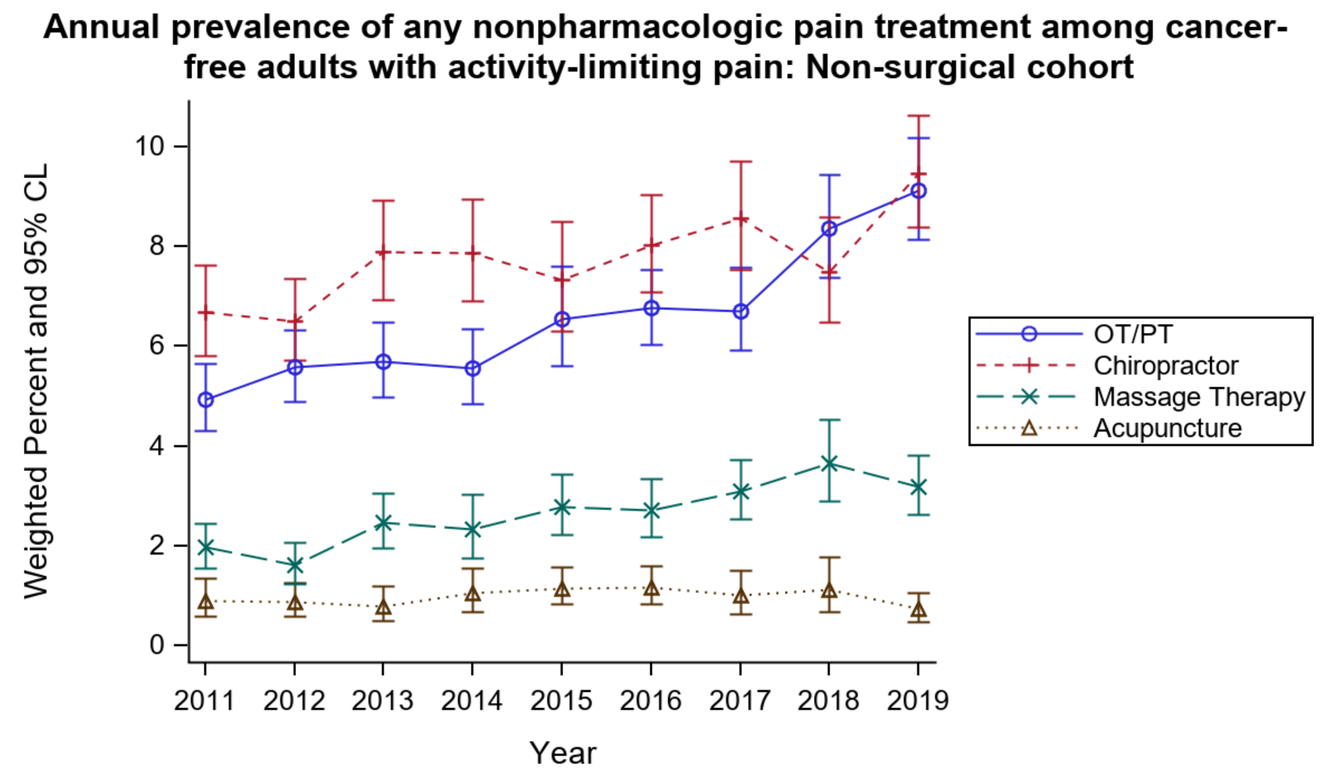

	2011	2012	2013	2014	2015	2016	2017	2018	2019
OT/PT	4.9	5.6	5.7	5.6	6.5	6.8	6.7	8.4	9.1
Chiropractor	6.7	6.5	7.9	7.9	7.3	8.0	8.6	7.5	9.5
Massage Therapy	2.0	1.6	2.5	2.3	2.8	2.7	3.1	3.6	3.2
Acupuncture	0.9	0.9	0.8	1.1	1.2	1.2	1.0	1.1	0.7

© 2022 Pritchard KT et al. *JAMA Network Open.*

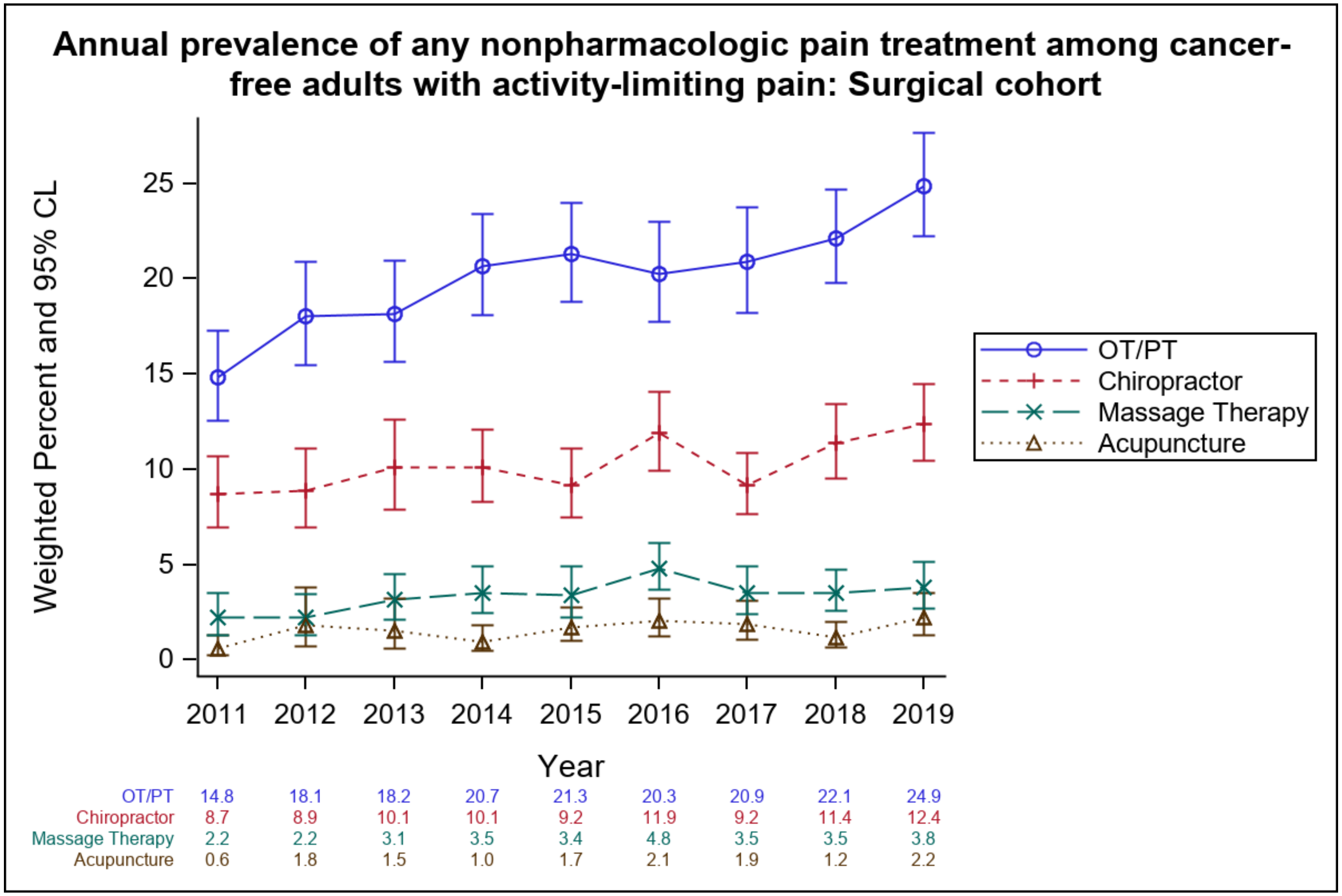

Note: 2011-2019 trends in the utilization of any nonpharmacologic pain treatment including acupuncture, chiropractic care, massage therapy, occupational therapy and/or physical therapy (OT/PT). Weighted estimates are reported with 95% Clopper Pearson confidence intervals.

© 2022 Pritchard KT et al. *JAMA Network Open.*

eTable 8. Weighted Multivariable Logistic Regression Analysis of Odds of Health Service Utilization among Cancer-free Adults with VR-12 Pain – MEPS, 2011-2019.

			Opioids Only	Nondrug Only	Both	Neither Treatment
		**Year**	**aOR (95% CI)**	**aOR (95% CI)**	**aOR (95% CI)**	**aOR (95% CI)**
Any VR-12 Pain	*Non-surgical Cohort n=64,062*	2011	REF	REF	REF	REF
		2012	1.20 (1.05-1.38)	0.81 (0.68-0.96)	1.20 (0.89-1.62)	0.98 (0.88-1.09)
		2013	1.13 (0.98-1.32)	1.20 (1.02-1.40)	1.63 (1.17-2.27)	0.80 (0.72-0.88)
		2014	1.31 (1.12-1.53)	1.04 (0.86-1.26)	1.79 (1.36-2.35)	0.78 (0.69-0.88)
		2015	1.16 (0.98-1.37)	1.08 (0.90-1.29)	1.71 (1.33-2.20)	0.82 (0.73-0.92)
		2016	0.77 (0.65-0.91)	1.19 (1.00-1.42)	1.10 (0.80-1.50)	1.01 (0.90-1.15)
		2017	0.89 (0.75-1.04)	1.16 (0.98-1.38)	1.40 (1.04-1.88)	0.93 (0.83-1.03)
		2018	0.84 (0.71-1.00)	1.25 (1.05-1.49)	1.36 (0.99-1.87)	0.91 (0.81-1.02)
		2019	0.65 (0.55-0.77)	1.45 (1.23-1.71)	1.14 (0.83-1.56)	0.94 (0.84-1.05)
		**Year**	**aOR (95% CI)**	**aOR (95% CI)**	**aOR (95% CI)**	**aOR (95% CI)**
	Surgical Cohort n=9,334	2011	REF	REF	REF	REF
		2012	1.15 (0.88-1.52)	1.03 (0.70-1.51)	1.30 (0.92-1.83)	0.82 (0.66-1.02)
		2013	1.25 (0.95-1.65)	1.05 (0.73-1.52)	1.35 (0.93-1.98)	0.76 (0.60-0.95)
		2014	1.06 (0.82-1.38)	1.31 (0.91-1.89)	1.87 (1.32-2.66)	0.68 (0.54-0.85)
		2015	1.28 (1.00-1.62)	0.96 (0.67-1.38)	1.81 (1.30-2.52)	0.68 (0.56-0.82)
		2016	0.85 (0.66-1.10)	1.02 (0.74-1.39)	1.73 (1.20-2.50)	0.90 (0.73-1.11)
		2017	0.89 (0.67-1.18)	0.94 (0.66-1.35)	1.50 (1.03-2.18)	0.95 (0.77-1.19)
		2018	0.84 (0.64-1.11)	1.07 (0.74-1.54)	1.69 (1.21-2.35)	0.89 (0.72-1.11)
		2019	0.84 (0.62-1.13)	1.39 (0.96-2.00)	1.63 (1.14-2.32)	0.82 (0.64-1.04)

Note: Adjusted for sociodemographic factors (age, sex, race, ethnicity, census region), socioeconomic status (income, education, insurance type), number of comorbidities (hypertension, coronary heart disease, emphysema, chronic bronchitis, diabetes, arthritis, asthma, stroke), and severity of pain interference. Mutually exclusive outcomes for opioid, non-pharmacologic, and both treatments in reference to those who used opioids alone.

Adjusted Odds Ratio (aOR); Confidence Interval (95% CI)

© 2022 Pritchard KT et al. *JAMA Network Open.*

eReferences

1. Tian TY, Zlateva I, Anderson DR. Using electronic health records data to identify patients with chronic pain in a primary care setting. *Journal of the American Medical Informatics Association.* 2013;20(e2):e275-e280.
2. Bernard D, Machlin, S.R., Fang, Z., and Cohen, J. Average Annual Opioid Use among Adults Treated for Conditions Associated with Chronic Pain versus Other Conditions 2013–2015. *Research Findings #43* 2019; https://meps.ahrq.gov/data_files/publications/rf43/rf43.pdf.
3. Encinosa W, Bernard D, Selden TM. Opioid and non-opioid analgesic prescribing before and after the CDC's 2016 opioid guideline. *International Journal of Health Economics and Management.* 2022;22(1):1-52.
4. Baillargeon J, Raji MA, Urban RJ, et al. Opioid-induced hypogonadism in the United States. *Mayo Clinic Proceedings: Innovations, Quality & Outcomes.* 2019;3(3):276-284.
5. Seal KH, Shi Y, Cohen G, et al. Association of mental health disorders with prescription opioids and high-risk opioid use in US veterans of Iraq and Afghanistan. *Jama.* 2012;307(9):940-947.
6. Hancox JG, Venkat AP, Coldiron B, Feldman SR, Williford PM. The safety of office-based surgery: review of recent literature from several disciplines. *Archives of Dermatology.* 2004;140(11):1379-1382.

© 2022 Pritchard KT et al. *JAMA Network Open.*
